# Measuring the Success of a Project ECHO Implementation: Results from an International e-Delphi Study

**DOI:** 10.1007/s43477-022-00050-7

**Published:** 2022-08-10

**Authors:** Perrin Moss, Nicole Hartley, Dana Newcomb, Trevor Russell

**Affiliations:** 1 Integrated Care, Children’s Health Queensland Hospital and Health Service, Brisbane, Australia; 2 School of Health and Rehabilitation Sciences, The University of Queensland, Brisbane, Australia; 3 School of Business, The University of Queensland, Brisbane, Australia; 4 Primary Care Clinical Unit, Faculty of Medicine, The University of Queensland, Brisbane, Australia; 5 RECOVER Injury Research Centre, The University of Queensland, Brisbane, Australia

**Keywords:** Project ECHO, Telementoring, Implementation, Innovation, Education, Workforce development

## Abstract

There is an increasing global need for organisations to utilise high-quality telementoring models to support workforce development and mentorship. Project ECHO is a validated telementoring model that has been adopted by over 700 organisations globally across multiple sectors. To date there is no consolidated list of success indicators by which organisational teams can assess or benchmark their implementation of Project ECHO across sectors. An e-Delphi methodology was adopted to facilitate a comprehensive means of identifying indicators that could be used to assess the implementations of Project ECHO globally. This paper presents a consolidated framework of indicators that support teams to assess their implementation of Project ECHO. These indicators have been derived by an international panel of experts across the healthcare, education, and university sectors. The final framework identified 54 distinct indicators across four domains: (1) spoke participant engagement, (2) ECHO Hub/teleECHO Network design and operation, (3) ECHO Hub team engagement and (4) Local Impact. This paper highlights that Project ECHO implementation indicators can vary between being dynamic, static, and iterative, depending on the phase of implementation. These findings are significant because they are generalisable to any organisation/sector implementing Project ECHO or similar telementoring models.

**Supplementary Information:**

The online version contains supplementary material available at 10.1007/s43477-022-00050-7.

## Introduction

Telementoring provides a platform for professionals with specific content knowledge and expertise within a particular organisation/context to collaborate, share and empower colleagues internally and externally (Agley et al., 2021; Bachynsky, 2020; Barbera et al., 2017; Christian & Andreas, 2019; Gegenfurtner & Ebner, 2019; Hauer & Quill, 2011). The virtual nature of telementoring overcomes traditional boundaries of geography, sector, professional discipline and seniority, and other siloes to integrate the way workforces can learn, seek advice and support, and consequently use newly acquired knowledge to enhance their service provision capabilities (Arora et al., 2020; De Witt Jansen et al., 2018; Gleason et al., 2020; Joshi et al., 2020; Katzman et al., 2020, 2021a, 2021b; Lewiecki et al., 2017; McPhillips et al., 2021; Nhung et al., 2021; Tosi et al., 2020). While telementoring is not a new concept, the contemporary implementation of telementoring models such as Project ECHO is a disruptive innovation which effects change within the organisation adopting the model for the first time. Organisations must be able to measure the change achieved by their implementation of telementoring innovations to benchmark for quality improvement and investment decision-making.

The COVID-19 pandemic has necessitated for many organisations to quickly adopt telementoring models to provide virtual learning and workforce development solutions in response to the rapidly changing landscapes across many sectors (Katzman et al., 2021a, 2021b). Telementoring emerged in the healthcare sector, evolving from telemedicine—or delivering direct patient care remotely via videoconferencing and teleconferencing (Singh et al., 2016), and the concept has since been replicated in a variety of non-healthcare sectors.

Project ECHO is a licensed and trademarked telementoring model which can be used to create virtual knowledge networks, or communities of practice, and incorporates case-based learning strategies from medical education and theoretical frameworks including Social Cognitive Theory, Situated Learning Theory, and Community of Practice Theory (Socolovsky et al., 2013; Wenger et al., 2002). The ECHO model™ was developed in 2003 by Professor Sanjeev Arora at the University of New Mexico (UNM) in the USA, as a platform for both improving healthcare service delivery and patient outcomes in treating Hepatitis C (Arora, Kalishman, et al., 2011). In 2011, UNM demonstrated that Project ECHO supported primary care providers to achieve equitable health outcomes in managing patients with Hepatitis C as those treated exclusively in tertiary hospital settings (Arora, Thornton, et al., 2011). It was highlighted that where geography prevented equitable access to high-quality care, in particular specialist care, the ECHO model overcame this barrier by connecting rural and remote providers with metropolitan-based experts (Arora, Thornton, et al., 2011). Thus, Project ECHO achieved positive health outcomes for patients by facilitating access to enhanced healthcare services conveniently in their local communities (Arora, Kalishman, et al., 2011).

Since the ECHO model’s initial uptake within the healthcare sector, it has grown to over 700 licensed organisations globally across 55 countries and beyond the original Hepatitis C focus to include an increasing variety of health, education and civic applications (ECHO Institute, 2020a). Diffusion of the ECHO model commences with organisational teams completing formal Immersion training, which is facilitated by one of several organisations designated as ECHO Superhubs under license by the UNM (ECHO Institute, 2019, 2020a). Immersion is a once-off compulsory training for prospective hub organisations to complete prior to becoming licensed to use the ECHO model. ECHO Superhubs are established and successful ECHO hub organisations which are designated by UNM to provide new organisational teams with Immersion training. Organisations who complete Immersion training are subsequently designated as ECHO hub sites and are licensed to replicate the ECHO model for the delivery of one or more ECHO networks as their local telementoring intervention. Superhubs are established ECHO hub organisations, which have an additional licensed function of providing Immersion training locally to actively support and mentor emerging teams from other organisations to understand how to implement the ECHO model. Superhubs support other organisational teams to understand how to implement the ECHO model with fidelity and integrate the telementoring intervention within business-as-usual processes to disrupt, innovate and improve outcomes regardless of their sectoral orientation.

The versatility of the ECHO model is evidenced through its growth and adoption across sectors other than healthcare including; business, corrections, domestic violence, education, ethics, law enforcement, quality improvement, social welfare, and more recently climate change and COVID-19 (ECHO Institute, 2020a). Fidelity to the ECHO model is universal (ECHO Institute, 2020b, 2020c), however there is no consolidated list of indicators by which teams can assess or benchmark their implementation across various sectors. This has prevented organisations from using the ECHO model to measure, compare and contrast implementation outcomes and experiences in a consistent way using universal indicators (Dearing et al., 2019). Since the emergence of COVID-19, rapid growth in organisations beyond healthcare adopting the ECHO model has occurred across a variety of sectors (ECHO Institute, 2021). This has created a gap in knowledge about what constitutes universal implementation success for organisational teams using the ECHO model globally in any sector (Dearing et al., 2019; Moss et al., 2021).

The strategies and tactics used by organisational teams implementing Project ECHO to provide telementoring solutions within their organisations may vary by sector (Moss et al., 2021). Despite strong evidence of the ECHO model being used in the healthcare sector, there is no published evidence of what universal indicators can assess the successful implementation of the telementoring model beyond healthcare (Arora, Thornton, et al., 2011; Zhou et al., 2016). It is important for organisations in any sector to be able to understand the level of success of their implementation and benchmark their ongoing use of the ECHO model for quality improvement and investment purposes. This research aimed to identify the indicators of implementation success and to develop a framework which could be scalable to any sector in which the ECHO model is used. The framework provides teams with a universal guide to measure their implementation, planning, and ongoing quality improvements of their local use of the ECHO model as a telementoring innovation.

## Methods

### Overview

The Delphi technique originated at the RAND Corporation in the 1950s and has proven to be a popular method to identify and prioritise issues for decision-making, with well-documented and stepwise frameworks to investigate research questions (Okoli & Pawlowski, 2004; Schmidt, 1997). The method has been described as a structured group communication process which facilitates answering a complex question through structured communication (Adler & Ziglio, 1996; Linstone & Turoff, 1975). The Delphi process includes the following key components: an opening question or prompt is offered; feedback of individual contributions of information and knowledge; assessment of the group judgement or view; opportunity for individuals to revise views; and anonymity for the individual responses (Linstone & Turoff, 1975). The Delphi technique was thus selected to obtain the most reliable consensus of implementation success indicators from across an international group of Project ECHO experts (Choi et al., 2019; Davies et al., 2021; Diamond et al., 2014; Fleuren et al., 2004; Garbutt et al., 2019).

Based on the established methodological approach for conducting Delphi studies, and the global distribution of ECHO hubs, a Delphi panel was established consisting of ECHO practitioners from international hub organisations and a five-round modified e-Delphi survey was conducted between May and December 2021 (Davies et al., 2021; Schmidt, 1997). This approach was pursued to achieve expert consensus to identify the indicators included in the framework that could assess and measure the successful implementation of the ECHO model within any organisational setting.

**Fig. 1:**
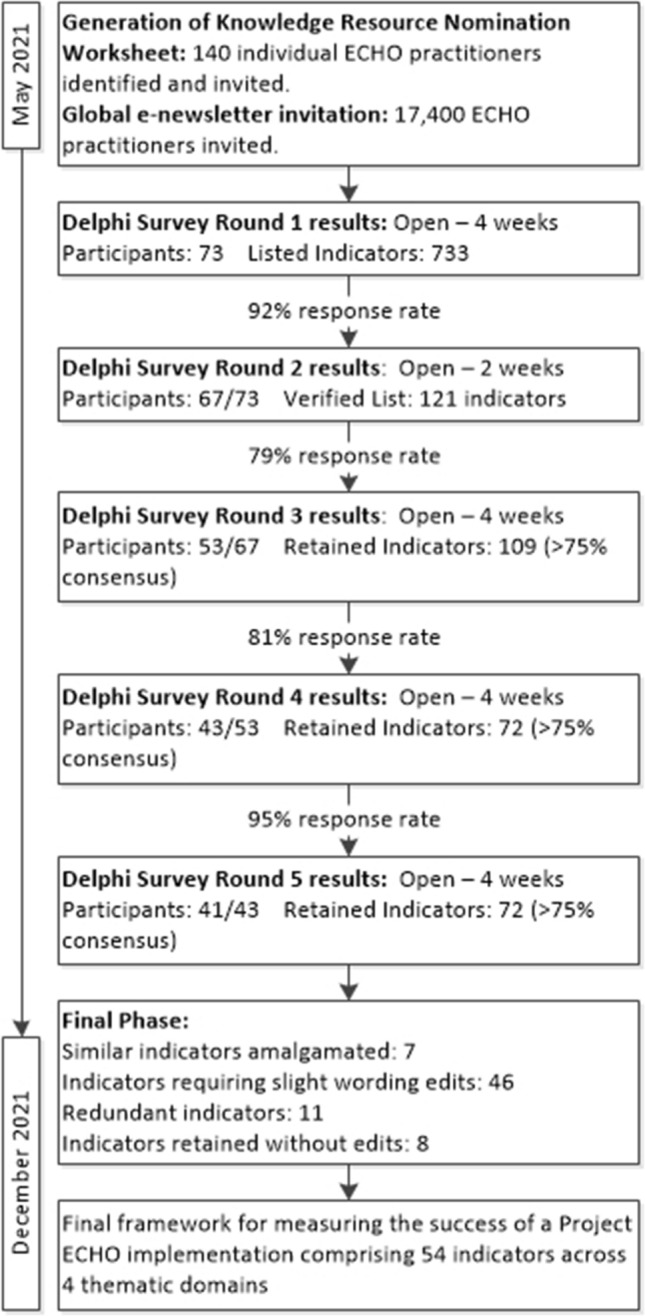
Phases of the e-Delphi study

The established methodological criteria for reporting Delphi study results were used to ensure the quality of this research (Adler & Ziglio, 1996; Egfjord & Sund, 2020; Fleuren et al., 2004; Haggar, 2018; Schmidt, 1997; Silva et al., 2018; Wild & Torgersen, 2000). Ethical approval was provided by both The University of Queensland and Children’s Health Queensland Hospital and Health Service’s Human Research Ethics Committees (LNR/21/QCHQ/75147 and SSA/2021/QCHQ/75147). Figure 1 below provides an outline of the e-Delphi study phases.

### Survey Development

#### Delphi Panel

An international panel of Project ECHO experts was established for the Delphi study to reach a consensus on the indicators of success of a Project ECHO implementation. A knowledge resource nomination worksheet (KRNW) was developed by the research team to identify an initial list of 140 individual ECHO practitioners from organisations with established ECHO hub and Superhub operations. The KRNW was informed by the academic, research, and ECHO network track records of these individuals gathered from a literature search and various open access Project ECHO websites (ECHO Institute, 2020a; Okoli & Pawlowski, 2004). An invitation to participate was sent to the 140 potential panel participants via email by the Principal Investigator (PM). A snowball method was used by inviting potential panellists to forward the invitation to their colleagues and broader network whom they believed would also be eligible. An open invitation to participate in the Delphi study was also included in the UNM’s ECHO Institute fortnightly newsletter which is distributed to a global readership of ECHO practitioners that exceeded 17,400. Inclusion criteria was defined as the Delphi participants must have completed Project ECHO Immersion training and/or have participated in the planning, coordination, and delivery of at least one ECHO Network. Interested prospective panellists completed a screening question at the beginning of the Round 1 e-Delphi survey to ensure they met the eligibility criteria. In subsequent rounds of the e-Delphi study, only those individuals who had completed the previous Delphi round were emailed the next iteration of the survey.

#### Ethical Considerations

Within this Delphi study, participants were able to present and react to ideas in a quasi-anonymous setting—while they were known to the Principal Investigator and perceivably to one another, their ideas, opinions, and rankings remained strictly anonymous throughout the study (Hasson et al., 2000).

#### e-Delphi Survey

The survey was administered via Qualtrics, an online survey platform, over five rounds. Each round was open for two-four weeks to maximise response rates, with weekly reminder emails sent during that time to non-responders to encourage them to complete the surveys. Each survey round took approximately 20–30 min to complete.

#### Round 1

In Round 1, panellists were asked how they would measure success of a Project ECHO implementation, by listing at least ten indicators they would consider useful. Panellists were also able to provide additional comments or recommendations if they wished. Prior to submitting their response to Round 1, informed consent was obtained from all individual panellists included in the study. The items contributed by panellists were then consolidated, with duplicates removed.

#### Round 2

In Round 2, panellists were provided with individualised reports mapping their Round 1 submissions to the consolidated list of indicators developed from the entire panel population. In this round, panellists were asked to verify if they agreed that their initial submissions had been fairly and accurately represented in the consolidated list (Schmidt, 1997).

#### Round 3

In Round 3, panellists were requested to rate each of the specific indicators in the consolidated list on a three-point scale of ‘unimportant’, ‘important’, or ‘essential’ for measuring the success of a Project ECHO implementation (Davies et al., 2021). The rationale for the three-point scale was to maximise panellist retention and to commence the rating process for the next round. Individual indicators which reached consensus amongst the panel were retained for further consideration in Round 4. In this round, consensus was defined as 75% of the panel agreeing that the indicator was either ‘important’ or ‘essential’ (Diamond et al., 2014).

#### Round 4

In Round 4, panellists were informed of indicators from Round 3 that had not reached consensus and were asked to rate those retained on an eleven-point numerical scale (with terminal anchors of 0 = strongly disagree and 10 = strongly agree) (Davies et al., 2021). The rationale for the eleven-point numerical scale was to elicit more specific ratings on the importance of each indicator. Indicators that achieved a consensus of at least 75% of the panel rating greater than 6 were retained for Round 5.

#### Round 5

In Round 5, panellists were asked to reconsider and re-rate the indicators from Round 4 using the same rating scale. To facilitate this process, each panellist was presented with their individual rating for each item in Round 4, with the overall panel mean rating for that item. Indicators that achieved a consensus of at least 75% of the panel rating greater than 7 were retained for inclusion in the final list of implementation success indicators.

#### Final Phase

In the final phase of this study, the entire research team (*n* = 4) reviewed and consolidated the final list of indicators retained from Round 5 during three one-hour consensus meetings. This involved the amalgamation of similar or overlapping indicators, removal of unmeasurable items, and slight rewording to optimise clarity. At this point, the entire research team collectively agreed that the thematic domains had emerged from the data and were used to group the final consolidated list of indicators as the final step. The final list approved was by the research team and distributed to all Delphi panellists to acknowledge the study’s data collection had concluded. These findings could be generalisable to any organisation/sector implementing Project ECHO to indicate the success of their implementation.

## Results

### Panellist Characteristics

The Delphi panellists were individuals working at ECHO hub organisations, who had completed Project ECHO Immersion training, and/or had participated in the planning, coordination, and delivery of at least one ECHO Network as a minimum. Table 1 describes the demographic characteristics of the Delphi panellists in this study. In Round 1, 73 participants from 36 ECHO hub organisations in 6 countries participated, with a significant proportion of females coming from healthcare and university organisations in Australia and the USA. In Round 2 there were 67 panellists retained, followed by 53 in Round 3, 43 in Round 4 and 41 in Round 5, representing a 92%, 79%, 81%, and 95% retention rate per round, respectively. The panellists that were not retained over the five rounds were also largely female, from healthcare and university organisations in Australia and the USA.

**Table 1:** Delphi Panel Demographics

Participants	Round 1	Round 2	Round 3	Round 4	Round 5
	(*n* = 73)	(*n* = 67)	(*n* = 53)	(*n* = 43)	(*n* = 41)
*Gender*					
Female	57	53	40	31	29
Male	15	14	13	12	12
Gender fluid	1				
*Organisation type*					
Consultancy	1	1	1	1	1
Healthcare	36	34	29	21	20
University (Healthcare & Education Faculties)	33	29	22	20	20
Professional body (Medical Education)	2	2	1	1	0
Information Technology	1	1	0	0	0
*Country*					
Australia	27	26	21	16	15
Canada	2	2	2	1	1
India	3	2	2	0	0
United Kingdom	3	3	3	3	3
USA	37	33	24	22	22
Zimbabwe	1	1	1	1	0

### Delphi Rounds 1–5

Figure 1 provides a summary of each round in the e-Delphi study that resulted in the development of the final framework with domains for the indicators of success (Appendix 1). In the first round, 733 individual indicators were submitted by 73 participants. The 733 indicators were then consolidated into a list of 121 randomly ordered indicators developed by the research team to remove duplicates, synthesise language and terminology (Appendix 2). In the second round, 67 retained panellists (92%) verified that they agreed that their initial submissions had been fairly and accurately represented in the consolidated list (Schmidt, 1997). The remaining 6 panellists (8%) did not respond to the Round 2 invitation. In the third round, 53 panellists (79%) and 109 indicators were retained, with 12 indicators (10%) not reaching consensus and were subsequently removed from the list (Appendix 3). In the fourth round, 43 panellists (81%) and 72 indicators were retained, with 37 indicators (34%) not reaching consensus that were subsequently removed from the list ahead of the final round (Appendix 4). In the fifth round, 41 panellists (95%) were retained, with all 72 indicators (100%) reaching consensus and were retained into the final phase. Data from Round 4 and 5 were analysed with SPSS software to determine the mean scores for each indicator and where consensus was achieved. Please note: all Appendices for this study are included in the supplementary files.

### Final Phase

When the final list of indicators from the Delphi panellists were reviewed by the research team, seven indicators were identified has having similar meanings, and as a result were amalgamated with other indicators (Appendix 5). Revised wording edits were made to 46 indicators for optimal clarity (Appendix 6). Eleven indicators were agreed to be redundant, with remaining indicators adequately capturing the same data (Appendix 7). Eight indicators were retained with no revised wording (Appendix 8).

The final list comprised 54 indicators of success that were aligned in the framework under four thematic domains illustrated in Table 2: (1) spoke participant engagement, (2) ECHO Hub/ECHO Network design and operation, (3) ECHO Hub team engagement and (4) Local Impact. Indicators of spoke participant engagement have been defined as those which measure the number, interactivity and participation experience of individuals who join ECHO Networks from a variety of spoke locations to connect and learn with panel teams centrally coordinated by the hub. Indicators of ECHO Hub/Network design and operation have been defined as those which measure the design and operation of an organisation’s ECHO Hub, and/or individual ECHO Networks. Indicators of ECHO Hub team engagement have been defined has those which measure the number, interactivity and participation experience of individuals who facilitate and manage ECHO Hub functions. Indicators of Local Impact have been defined as those which measure the increase or improvement in workforce development, capacity, system integration and efficiency. The four thematic domains were identified through an inductive thematic approach to group individual indicators to target specific areas of implementation success identified by the panellists.

**Table 2:** Thematic domains and indicators of implementation success for project ECHO—framework (N = 54)

#	Indicator of success	Recommended measurement phase (pre-launch, launch, growth/continuous improvement)	Data collection point/stakeholder (individual spoke participant/panellist, ECHO network, ECHO hub, organisation, system)	Recommended method of measurement
*Domain 1: spoke participant engagement—14 indicators* Definition: Indicators which measure the number, interactivity and participation experience of individuals who join ECHO Networks from a variety of spoke locations to connect and learn with panel teams centrally coordinated by the hub				
1.1	Spoke participants attend teleECHO Network sessions regularly	Launch, Growth/ Continuous Improvement	Individual Spoke Participant	iECHO CRM teleECHO clinic attendance report: individual participant attendance
1.2	Spoke participant diversity (gender, profession, culture, geography) attendance which meets target numbers	Launch, Growth/ Continuous Improvement	Individual Spoke Participant	iECHO CRM teleECHO clinic attendance report: individual participant attendance
1.3	Evidence of peer-to-peer testimonials	Growth/ Continuous Improvement	Individual Spoke Participant	Documentation of/recordings of testimonials, Spoke Participant Surveys (individuals), Single Session Feedback (polling, surveys), Interviews and Focus Groups
1.4	Higher levels of spoke participant experience (enjoyable, collegial, inclusive, non-judgemental)	Growth/ Continuous Improvement	Individual Spoke Participant	Spoke Participant Surveys (individuals), Single Session Feedback (polling, surveys), Interviews and Focus Groups
1.5	Number of spoke participants who present cases for discussion	Growth/ Continuous Improvement	Individual Spoke Participant	iECHO CRM participant report, iECHO CRM teleECHO clinic report
1.6	Number of ECHO sessions where spoke participants present cases from their local context	Growth/ Continuous Improvement	ECHO Network	iECHO CRM participant report, iECHO CRM teleECHO clinic report
1.7	Higher levels of spoke participant safety and comfort in volunteering to present cases from their own context as a learning opportunity within the teleECHO Network	Growth/ Continuous Improvement	Individual Spoke Participant	Spoke Participant Surveys (individuals), Single Session Feedback (polling, surveys), Interviews and Focus Groups, iECHO CRM teleECHO clinic report
1.8	Higher levels of spoke participant satisfaction with didactic content, panel expert(s) representation/hub team support	Growth/ Continuous Improvement	Individual Spoke Participant	Spoke Participant Surveys (individuals), Single Session Feedback (polling, surveys), Interviews and Focus Groups, iECHO CRM teleECHO clinic report
1.9	Higher levels of spoke participant satisfaction with learning/advice/support gained from case presentation and discussion (applies to individual case presenter, as well as other spoke participants learning from the case) and recommendations	Growth/ Continuous Improvement	Individual Spoke Participant	Spoke Participant Surveys (individuals), Single Session Feedback (polling, surveys), Interviews and Focus Groups
1.10	Number of spoke participants who represent cases	Launch, Growth/ Continuous Improvement	Individual Spoke Participant	iECHO CRM participant report, iECHO CRM teleECHO clinic report
1.11	Higher levels of spoke participant satisfaction with the opportunity to contribute to the dialogue, ask questions, make recommendations whether verbally or non-verbally	Growth/ Continuous Improvement	Individual Spoke Participant	Spoke Participant Surveys (individuals), Single Session Feedback (polling, surveys), Interviews and Focus Groups
1.12	High reported levels of spoke participants self-reporting that they feel safe, supported, and welcomed at teleECHO Network sessions	Growth/ Continuous Improvement	Individual Spoke Participant, ECHO Network	Spoke Participant Surveys (individuals), Single Session Feedback (polling, surveys), Interviews and Focus Groups, iECHO CRM teleECHO clinic report
1.13	Measurable increase in spoke participants who contribute to the discussion verbally or via chat	Growth/ Continuous Improvement	ECHO Network	teleECHO Scorecard
1.14	Evidence of spoke participants inviting colleagues to attend teleECHO Network sessions to co-present case presentations	Growth/ Continuous Improvement	Individual Spoke Participant, ECHO Network	iECHO CRM participant report, iECHO CRM teleECHO clinic report
*Domain 2: ECHO Hub/teleECHO Network design and operation—23 indicators* Definition: Indicators which measure the design and operation of an organisation’s ECHO Hub, and/or individual ECHO Networks				
2.1	Evidence of the teleECHO Network's co-design occurred with prospective participants, consumers, system managers, and subject matter experts	Pre-Launch, Growth/ Continuous Improvement	ECHO Network	Review of Implementation plan, Evaluation plan, Learning Needs Assessment results
2.2	Number of discrete stakeholders involved in the co-design of the teleECHO Network	Pre-Launch, Growth/ Continuous Improvement	ECHO Network	Review of Implementation plan, Evaluation plan, Learning Needs Assessment results
2.3	Demonstrated alignment to local, state, federal priorities, and associated quality/funding metrics	Pre-Launch, Growth/ Continuous Improvement	ECHO Network, ECHO Hub, Organisation, System	Review of Implementation plan, Evaluation plan, Learning Needs Assessment results, and teleECHO Network funding sources
2.4	Evidence that the teleECHO Network delivers on the findings of the learning needs assessment	Growth/ Continuous Improvement	ECHO Network, ECHO Hub	Review of Implementation plan, Evaluation plan, Learning Needs Assessment results, Spoke Participant Surveys (individuals), Single Session Feedback (polling, surveys), Interviews and Focus Groups
2.5	Measurable increase in levels of interactivity amongst spoke participants and panellists during sessions (on camera, chat, verbal, non-verbal, volunteering to present cases)	Growth/ Continuous Improvement	ECHO Network—Spoke Participants, Panellists	teleECHO Scorecard
2.6	Frequency/ regularity of sessions—sessions are held routinely	Launch, Growth/ Continuous Improvement	ECHO Network	iECHO CRM participant report, iECHO CRM teleECHO clinic report
2.7	Higher levels of balance in dialogue contributed by panellists vs spoke participants, demonstrating spokes are contributing at least 50% of the talking	Growth/ Continuous Improvement	ECHO Network	teleECHO Scorecard
2.8	Number of teleECHO sessions including a participant case	Launch, Growth/ Continuous Improvement	ECHO Network	iECHO CRM participant report, iECHO CRM teleECHO clinic report
2.9	High levels of teleECHO sessions being a non-hierarchical, professional forum for knowledge sharing is fostered by panellists	Launch, Growth/ Continuous Improvement	Individual ECHO Network Panellist, ECHO Network	teleECHO Scorecard, Spoke Participant Surveys (individuals), Single Session Feedback (polling, surveys), Interviews and Focus Groups
2.10	Evidence of streamlined hub operational and logistical processes that optimise the delivery of teleECHO sessions	Pre-Launch, Growth/ Continuous Improvement	ECHO Network, ECHO Hub	teleECHO Scorecard, iECHO CRM participant report, iECHO CRM teleECHO clinic report, evidence of localised policies, procedures, manuals for ECHO hub operations
2.11	Evidence that the teleECHO Panel adheres to the Anatomy of an ECHO for fidelity assurance	Launch, Growth/ Continuous Improvement	Individual ECHO Network Panellist, ECHO Network	teleECHO Scorecard, Spoke Participant Surveys (individuals), Single Session Feedback (polling, surveys), Interviews and Focus Groups
2.12	Evidence of ECHO hub teams undertaking learner needs assessment, implementation planning, evaluation planning, panel expertise onboarding, and 2 mock ECHO sessions prior to launching a teleECHO Network	Pre-Launch	Individual ECHO Network Panellist, ECHO Hub	Review of Implementation plan, Evaluation plan, Learning Needs Assessment results, Interviews and Focus Groups, mock and post-launch teleECHO Scorecards
2.13	Evidence of hub team attracting sufficient funding to fulfil implementation/ hub management/ replication functions sustainably	Pre-Launch, Launch, Growth/ Continuous Improvement	ECHO Hub	Organisational cost centre/financial reports
2.14	Evidence of hub team managing operations within budget constraints of the organisation	Launch, Growth/ Continuous Improvement	ECHO Hub	Organisational cost centre/financial reports
2.15	Evidence of ECHO hub leadership role(s) and clear organisational governance oversight of ECHO hub team structure are present	Pre-Launch, Launch, Growth/ Continuous Improvement	ECHO Hub	ECHO team role descriptions, organisational structure, organisation's operational and/or strategic plans
2.16	Evidence of an interprofessional and diverse hub team	Pre-Launch, Launch, Growth/ Continuous Improvement	ECHO Hub	ECHO team role descriptions, organisational structure
2.17	Evidence of communication systems/processes developed for routine engagement with stakeholders outside of teleECHO sessions	Launch, Growth/ Continuous Improvement	Individual ECHO Network Panellist, ECHO Hub	Correspondence records, mailing lists, templates/CRM for distribution of didactic resources, reference lists, journal articles, podcasts, and other resources
2.18	Evidence of hub team’s development and dissemination of marketing materials to increase awareness of and attraction to their ECHO operations	Pre-Launch, Launch, Growth/ Continuous Improvement	Individual ECHO Network Panellist, ECHO Hub	Media analytics dashboards that can be tailored/shared widely across multiple stakeholder audiences as appropriate including content attesting to the quality/credibility of organisational hub team/panellists. Examples would include succinct and engaging marketing materials to increase awareness of and attraction to ECHO activities and be tailored/shared widely across multiple audiences
2.19	Evidence of panellists and spoke participants advocating via word of mouth, peer-to-peer, personal/ professional network communication/ recommendations about joining teleECHO network(s)	Pre-Launch, Launch, Growth/ Continuous Improvement	Individual Spoke Participant, Individual ECHO Network Panellist, ECHO Network	Qualitative documentation of/recordings of testimonials, Spoke Participant Surveys (individuals), Single Session Feedback (polling, surveys), Interviews and Focus Groups
2.20	Evidence of hub stakeholders (champion, facilitator, panellists, coordinator) completing ECHO Immersion training provided by a designated Superhub prior to launch	Pre-Launch	Individual ECHO Network Panellist, ECHO Hub	Immersion attendance records
2.21	Evidence of ECHO hub team engaging with Superhub for post-Immersion partner liaison support and mentorship	Pre-Launch, Launch, Growth/ Continuous Improvement	Individual ECHO Network Panellist, ECHO Hub	iECHO/Salesforce CRM reports (partner liaison, technical assistance)
2.22	Evidence of hub teams having data collection processes to ensure all pertinent data is collected and evaluated in a reliable way	Pre-Launch, Launch, Growth/ Continuous Improvement	ECHO Network Panel, ECHO Hub	teleECHO Scorecard records, iECHO CRM reports, evidence of localised protocols for ECHO hub data collection and evaluation
2.23	Evidence of executive/ leadership support—where ECHO activities strategically align to organisational priorities, funding/investment decision-making	Pre-Launch, Launch, Growth/ Continuous Improvement	ECHO Hub, Organisation	Qualitative and quantitative documentation of/recordings of executive/leadership endorsement/advocacy for ECHO, reference points in organisational strategies, policies, plans, financial cost centre reports
*Domain 3: ECHO Hub team engagement—5 indicators* Definition: Indicators which measure the number, interactivity and participation experience of individuals who facilitate and manage ECHO Hub functions				
3.1	High levels of panellist experience and satisfaction (enjoyable, high value, time efficient)	Growth/ Continuous Improvement	Individual ECHO Panellist, ECHO Network	Panellist Surveys (individuals), Single Session Feedback (polling, surveys), Interviews and Focus Groups
3.2	Evidence of relevant stakeholders having a clear understanding of the ECHO model, its theoretical and practical application, and potential benefits	Launch, Growth/ Continuous Improvement	Individual ECHO Network Panellist, ECHO Hub, Organisation	Review of Implementation plan, Evaluation plan, Learning Needs Assessment results, Interviews and Focus Groups
3.3	High levels of strong and organised facilitation role/function, panel cohesion and satisfaction during teleECHO Network sessions	Launch, Growth/ Continuous Improvement	Individual ECHO Network Panellist, ECHO Network	teleECHO Scorecard, evidence of localised policies, procedures, manuals for ECHO hub operations
3.4	Demonstrated ability to recruit and retain Champion, Facilitator, Panellists with the right qualification, skills, expertise, lived experience, ability to present well and make spoke participants feel comfortable	Launch, Growth/ Continuous Improvement	Individual ECHO Network Panellist, ECHO Network	teleECHO Scorecard, ECHO team role descriptions
3.5	High levels of panel facilitator and panellist satisfaction with learning/ advice/ support contributed to/ arising from case presentation/s (applies to panel contributions, individual case presenter, as well as other spoke participants' learning and contribution to recommendations for the case/s)	Launch, Growth/ Continuous Improvement	Individual ECHO Network Panellist, ECHO Network	Panellist Surveys (individuals), Single Session Feedback (polling, surveys), Interviews and Focus Groups
*Domain 4: Local Impact—12 indicators* Definition: Indicators which measure the increase or improvement in workforce development, capacity, system integration and efficiency				
4.1	Measurable increase in spoke participant's confidence to manage cases locally	Pre-Launch, Growth/ Continuous Improvement	Individual Spoke Participant	Spoke Participant Surveys (individuals), Single Session Feedback (polling, surveys), Interviews and Focus Groups
4.2	Measurable increase in spoke participant's competence to manage cases locally	Pre-Launch, Growth/ Continuous Improvement	Individual Spoke Participant	Spoke Participant Surveys (individuals), Single Session Feedback (polling, surveys), Interviews and Focus Groups
4.3	Measurable increase in spoke participant's knowledge/skills to manage cases locally	Pre-Launch, Growth/ Continuous Improvement	Individual Spoke Participant	Spoke Participant Surveys (individuals), Single Session Feedback (polling, surveys), Interviews and Focus Groups
4.4	Measurable increase in spoke participant's capacity to manage cases locally	Pre-Launch, Growth/ Continuous Improvement	Individual Spoke Participant	Spoke Participant Surveys (individuals), Single Session Feedback (polling, surveys), Interviews and Focus Groups
4.5	Measurable increase in spoke participant self-reported change in experience to become a local expert to whom colleagues in their community/proximity refer to and collaborate with for support on cases	Pre-Launch, Growth/ Continuous Improvement	Individual Spoke Participant	Spoke Participant Surveys (individuals), Single Session Feedback (polling, surveys), Interviews and Focus Groups
4.6	Spoke participants applying of at least one change in their practice due to their participation in teleECHO Networks	Growth/ Continuous Improvement	Individual Spoke Participant	Spoke Participant Surveys (individuals), Single Session Feedback (polling, surveys), Interviews and Focus Groups
4.7	Measurable increase in spoke participant self-efficacy	Pre-Launch, Growth/ Continuous Improvement	Individual Spoke Participant	Spoke Participant Surveys (individuals), Single Session Feedback (polling, surveys), Interviews and Focus Groups
4.8	Measurable reduction in spoke participant's sense of professional isolation	Pre-Launch, Growth/ Continuous Improvement	Individual Spoke Participant	Spoke Participant Surveys (individuals), Single Session Feedback (polling, surveys), Interviews and Focus Groups
4.9	Measurable increase in spoke participant's joy of work	Pre-Launch, Growth/ Continuous Improvement	Individual Spoke Participant	Spoke Participant Surveys (individuals), Single Session Feedback (polling, surveys), Interviews and Focus Groups
4.10	Higher spoke participant reported positive changes in knowledge-sharing relationships between colleagues locally	Growth/ Continuous Improvement	Individual Spoke Participant, ECHO Network	Spoke Participant Surveys (individuals), Single Session Feedback (polling, surveys), Interviews and Focus Groups
4.11	Improvements in service utilisation, service wait times, distance travelled to access services by patients/consumers/ clients	Launch, Growth/ Continuous Improvement	Individual consumer, Individual ECHO Spoke Participant, ECHO Network, ECHO Hub, Organisation, System	teleECHO case presentation and patient/client record audits, postcode mapping, economic modelling, and analysis
4.12	Improvements in spoke participant’s professional relationships, access to specialist services, referral pathways, informed decision-making, peer-to-peer supports outside of teleECHO sessions which impact their patient/client care/service provision/professional isolation	Launch, Growth/ Continuous Improvement	Individual Spoke Participant	Qualitative documentation of/recordings of testimonials, Spoke Participant Surveys (individuals), Single Session Feedback (polling, surveys), Interviews and Focus Groups. Social Network Analyses

The framework provides recommendations for when each indicator be measured (Pre-Launch, Launch, Growth/Continuous Improvement) which is consistent with the three phases of replicating the ECHO model in any organisation (Children's Health Queensland Hospital and Health Service, 2021). Pre-Launch is the preparatory phase of implementation following the completion of Immersion training prior to the launch of a hub’s first ECHO Network. The Launch phase is the period encompassing the actual launch and first iteration of a hub’s pilot ECHO Network. The Growth/Continuous Improvement phase refers to the continuous period beyond the hub’s first ECHO Network, where the hub team undertake growth and continuous improvement activities within their hub.

The indicators are also aligned to which stakeholder group they relate to (Individual Spoke Participant/Panellist, ECHO Network, ECHO Hub, Organisation, System). To provide organisational teams implementing the ECHO model with consistent methods by which to measure each of the indicators with fidelity, recommended approaches have also been included. Using the framework, teams can analyse changes that occur over the lifecycle of their implementation with some indicators varying between being dynamic, static, and iterative—meaning that there are indicators which can be measured at random, as a once-off, or routinely. The variety of indicators highlights that implementation teams can capture learnings about their journey’s successes and/or areas for improvement based on what is of most interest/relevance. Indicators in this framework lend themselves to being measured or analysed at random, once-off, or routine milestones which will support ECHO practitioners to report on their successes at milestones throughout their implementation journey for quality improvement and ongoing investment opportunities. The detailed version of this final framework is included in Table 2.

## Discussion

The aim of this study was to identify indicators of implementation success using an e-Delphi approach, and compile them in an internationally relevant framework to measure and assess the implementation success of the ECHO model within any organisation. The purpose and intention of this framework is to provide structure and a frame of reference to support planning and decision-making (Cash-Gibson et al., 2019; Davies et al., 2021). The final framework comprised 54 specific indicators that were mapped across four thematic domains following the e-Delphi study. This framework provides a clearly articulated list of indicators required by ECHO practitioners to evaluate if their local implementation of the ECHO model is successful. The intention of this framework was to compile the indicators identified during the e-Delphi study, by global ECHO practitioners with expertise in implementing the ECHO model, under key thematic domains accompanied with recommendations for measurement. This provides ECHO practitioners with a consistent approach by which to measure each of the indicators in any sector with fidelity when assessing the implementation milestones within their organisation.

Globally, the rapid uptake of virtual telementoring models like Project ECHO has coincided with the onset of the COVID-19 pandemic (ECHO Institute, 2021). In 2019, there was approximately 232,000 ECHO session attendances by spoke participants, which grew to 1,162,000 in 2020. This represented a five-fold upswing in the utilisation of virtual solutions for workforce development and mentorship (ECHO Institute, 2021). The rapid increase in utilisation justified a need to swiftly develop this framework to support emerging and existing ECHO practitioners and implementation teams to understand what indicators could measure implementation success in any organisation or sector. Given there is a vacuum in the literature for guidance on what constitutes implementation success for Project ECHO, this study consolidated expertise garnered from international ECHO practitioners with cumulative experience and understanding of the ECHO model across a variety of contexts to identify the indicators in this framework for wider use.

To date, no previous studies have examined what ECHO practitioners and implementation teams consider to be universal indicators of implementation success for telementoring innovations like Project ECHO, particularly beyond the healthcare sector. These findings complement and build on other previously developed organisational readiness and implementation process tools/frameworks for ECHO practitioners at the beginning of their implementation phase (Serhal et al., 2018). One key point of difference with this study’s findings is that these domains and indicators have been elicited by ECHO practitioners across a variety of sectors (healthcare, education, university, public and private) where the ECHO model remains in use as a telementoring solution. This suggests that the framework may be relevant beyond the traditional context of many ECHO telementoring solutions operating within the healthcare sector.

This framework presents the most important indicators that need to be considered when implementing the ECHO model within organisations as determined by seasoned ECHO practitioners and implementation teams from across the globe. Further research investigating the application of this framework within ECHO hubs globally may also provide valuable insights to inform ongoing quality improvement efforts of hub organisations. This would provide ECHO Superhub training teams with a better understanding and response to the training and support needs of new ECHO hub teams. This may lead to more successful implementations globally. While this framework has been developed with the ECHO model at front of mind, it could be easily adopted for use for other innovative telementoring-like solutions being implemented by organisational teams. It has global relevance for adoption by a range of stakeholders, including ECHO practitioners, implementation teams, executive decision-makers, and other system managers across multiple sectors.

The framework domains captured in Table 2 of Spoke participant engagement, ECHO Hub/ECHO Network design and operation, ECHO Hub team engagement and Local Impact, provides a foundation for ECHO practitioners and implementation teams with a universal framework to consistently assess and benchmark their local successes across any organisational, sectoral, or geographic context. The framework provides organisational teams with consistent methods to measure each of the 54 indicators over the duration of their implementation. Indicators in this framework lend themselves to being measured or analysed at random, once-off, or routine milestones which will support ECHO practitioners to report on their successes at milestones throughout their implementation journey and benchmark against other global hubs. This framework might also be used by executive decision-makers and investors to inform how they organisationally commit to and financially invest in the ECHO model (Moss et al., 2020).

Since uptake of the ECHO model has expanded beyond the healthcare sector, this framework offers guidance and support for new and existing ECHO hubs as the model’s global diffusion continues. This framework also offers support for designated ECHO Superhubs to enhance their Immersion training curricula as it integrates with the universal phases of replicating the ECHO model (Pre-Launch, Launch, Growth/Continuous Improvement) (Children's Health Queensland Hospital and Health Service, 2021; ECHO Institute, 2019). Implementation teams could benefit from adopting this framework at Immersion training to enhance their pre-launch phase understanding of what indicates implementation success of the ECHO model in their local organisational context. This framework could facilitate enhanced consistency in reporting across ECHO hub teams’ implementation and evaluation planning activities by using these indicators and recommended measures. The framework highlights which indicators relate to specific stakeholder groups so implementation teams can collect and report the most meaningful data that indicates success for that audience (Individual Spoke Participant/Panellist, ECHO Network, ECHO Hub, Organisation, System) to measure their impact. This framework could be harnessed by ECHO hub teams and other telementoring researchers globally to plan, measure and showcase the changes achieved by their implementation of telementoring innovations using these indicators as consistent benchmarks for quality improvement and investment decision-making.

A strength of this study is the inclusion of a large e-Delphi panel with acceptable rates of panellist retention. The panel included 73 experts and retained 41 (56%) throughout the five survey rounds. Despite there not being a recommended or ideal number of panellists in a Delphi study, it is typical for panels to range in size between 10 and 100 (Diamond et al., 2014; Hasson et al., 2000; Kidholm et al., 2018; Snyder-Halpern, 2001). Given there were 36 ECHO hubs represented in this study out of approximately 700 active global hubs, this Delphi panel might be considered large. A second strength was that this Delphi had a global reach and demographic diversity, comprising experts from across six countries and five broad organisational types. This breadth in representation will ensure that the framework is internationally relevant.

Due to the high number of participants from the healthcare sector, the authors acknowledge there may be some residual desirability bias. Some indicators of success that can more easily be measured and reported on by teams using the ECHO model within the healthcare sector may have been ranked consistently higher than others (Ecken et al., 2011). The authors acknowledge that this study did not explore the correlation between the measures of implementation success that were identified during the e-Delphi with actual measurement of outcomes or impact. A subsequent research study is in train to investigate the application of the framework in a variety of organisational and sectoral contexts to test the framework.

## Conclusion

The results of this international e-Delphi study have been presented as a framework of indicators to support organisational teams to measure the success of their implementation of Project ECHO. The framework consolidated 54 distinct indicators which could be generalisable to any organisation/sector implementing Project ECHO to illustrate and showcase the successes or areas for improvement of local implementations of the ECHO model or other new telementoring innovations globally. By framing the indicators under four key domains, ECHO practitioners and implementation teams can consistently assess and benchmark their local successes or failures regardless of organisational, sectoral, or geographic context. The findings of this study are translational for audiences from other countries and sectors to indicate successful implementations and benchmark for ongoing quality improvement and investment in the organisation’s use of the ECHO model or other telementoring innovations.

## Supplementary Information

Below is the link to the electronic supplementary material.

Supplementary file1 (DOCX 58 kb)

## Data Availability

The authors plan to make anonymised data accessible to those who request it via email.

## References

[CR1] Adler, M., & Ziglio, E. (Eds.). (1996). Gazing into the oracle: The Delphi method and its application to social policy and public health. Jessica Kingsley Publishers.

[CR2] Agley J, Delong J, Janota A, Carson A, Roberts J, Maupome G (2021). Reflections on Project ECHO: Qualitative findings from five different ECHO programs. Medical Education Online.

[CR3] Arora S, Kalishman S, Dion D, Som D, Thornton K, Bankhurst A (2011). Partnering urban academic medical centers and rural primary care clinicians to provide complex chronic disease care. Health affairs (Project Hope).

[CR4] Arora S, Mate KS, Jones JL, Sevin CB, Clewett E, Langley G (2020). Enhancing collaborative learning for quality improvement: Evidence from the improving clinical flow project, a breakthrough series collaborative with project ECHO. Joint Commission Journal on Quality and Patient Safety.

[CR5] Arora S, Thornton K, Murata G, Deming P, Kalishman S, Dion D (2011). Outcomes of treatment for hepatitis C virus infection by primary care providers. The New England Journal of Medicine.

[CR6] Bachynsky N (2020). Implications for policy: The Triple Aim, Quadruple Aim, and interprofessional collaboration. Nursing Forum.

[CR7] Barbera E, Garcia I, Fuertes-Alpiste M (2017). A co-design process microanalysis: Stages and facilitators of an inquiry-based and technology-enhanced learning scenario. International Review of Research in Open and Distance Learning.

[CR8] Cash-Gibson L, Tigova O, Alonso A, Binkley G, Rosenmöller M (2019). Project INTEGRATE: Developing a framework to guide design, implementation and evaluation of people-centred integrated care processes. International Journal of Integrated Care.

[CR9] Children's Health Queensland Hospital and Health Service. (2021). *Start an ECHO and partner with us. Queensland Government*. Retrieved 19/01/2022 from https://echo.qld.gov.au/start-an-echo

[CR10] Choi WS, Park J, Choi JYB, Yang J-S (2019). Stakeholders' resistance to telemedicine with focus on physicians: Utilizing the Delphi technique. Journal of Telemedicine and Telecare.

[CR11] Christian E, Andreas G (2019). Learning and satisfaction in webinar, online, and face-to-face instruction: A meta-analysis. Frontiers in Education.

[CR12] Davies L, Hinman RS, Russell T, Lawford B, Bennell K, Billings M (2021). An international core capability framework for physiotherapists to deliver quality care via videoconferencing: A Delphi study. Journal of Physiotherapy.

[CR13] De Witt Jansen B, Brazil K, Passmore P, Buchanan H, Maxwell D, McIlfatrick SJ (2018). Evaluation of the impact of telementoring using ECHO© technology on healthcare professionals’ knowledge and self-efficacy in assessing and managing pain for people with advanced dementia nearing the end of life. BMC Health Services Research.

[CR14] Dearing, J. W., Cruz, S., Kee, K., Larson, R. S., & Rahm, A. K. (2019). *Project ECHO review and research agenda*.

[CR15] Diamond IR, Grant RC, Feldman BM, Pencharz PB, Ling SC, Moore AM, Wales PW (2014). Defining consensus: A systematic review recommends methodologic criteria for reporting of Delphi studies. Journal of Clinical Epidemiology.

[CR16] ECHO Institute. (2019). *Project ECHO (Extension for Community Healthcare Outcomes) Glossary of Terms*. https://echo.unm.edu/data/glossary

[CR27] ECHO Institute. (2020a). *ECHO hubs*. University of New Mexico Health Sciences Center. https://echo.unm.edu/locations

[CR18] ECHO Institute. (2020b). *ECHO Overview Infographic (2-page)*. University of New Mexico Health Sciences Center. Retrieved 11 February 2020 from https://echo.unm.edu/data/two-pager

[CR28] ECHO Institute. (2020c). *Project ECHO*. University of New Mexico Health Sciences Centre. Retrieved February 11, 2020c from https://echo.unm.edu/

[CR29] ECHO Institute. (2021). *ECHO movement overview*. University of New Mexico. Retrieved November 17, 2021 from https://hsc.unm.edu/echo/partner-portal/data-marketplace/interactive-dashboards/movement-overview.html

[CR17] Ecken P, Gnatzy T, von der Gracht HA (2011). Desirability bias in foresight: Consequences for decision quality based on Delphi results. Technological Forecasting & Social Change.

[CR19] Egfjord KF-H, Sund KJ (2020). Do you see what I see? How differing perceptions of the environment can hinder radical business model innovation. Technological Forecasting & Social Change.

[CR20] Fleuren M, Wiefferink K, Paulussen T (2004). Determinants of innovation within health care organizations Literature review and Delphi study. International Journal of Quality Health Care.

[CR21] Garbutt J, Antes A, Mozersky J, Pearson J, Grailer J, Toker E, DuBois J (2019). Validating curricular competencies in innovation and entrepreneurship for biomedical research trainees: A modified Delphi approach. Journal of Clinical and Translational Science.

[CR22] Gegenfurtner A, Ebner C (2019). Webinars in higher education and professional training: A meta-analysis and systematic review of randomized controlled trials. Educational Research Review.

[CR23] Gleason LJ, Beiting KJ, Walker J, Shervani S, Graupner J, Mittal K (2020). Using telementoring to share best practices on COVID-19 in post-acute and long-term care facilities. Journal of the American Geriatrics Society (JAGS).

[CR24] Haggar, F. L. (2018). *Innovation in health science education: A Delphi study of insights from experts in the field*. In: ProQuest Dissertations Publishing.

[CR25] Hasson F, Keeney S, McKenna H (2000). Research guidelines for the Delphi survey technique. Journal of Advanced Nursing.

[CR26] Hauer J, Quill T (2011). Educational needs assessment, development of learning objectives, and choosing a teaching approach. Journal of Palliative Medicine.

[CR30] Joshi S, Gali K, Radecki L, Shah A, Hueneke S, Calabrese T (2020). Integrating quality improvement into the ECHO model to improve care for children and youth with epilepsy. Epilepsia.

[CR31] Katzman J, Gygi K, Swift R, Comerci G, Bhatt S, Daitz B (2020). How hands-on pain skills intensive trainings complement ECHO pain and opioid management programs: A program evaluation with the Indian Health Service. Pain Medicine.

[CR32] Katzman J, Herring D, Schramm P, Tomedi L, Maury J-M, Kalishman S (2021). Climate Change and Human Health ECHO: Global Telementoring for Health Professionals..

[CR33] Katzman J, Thornton K, Sosa N, Tomedi L, Hayes L, Sievers M (2021). Educating health professionals about COVID-19 with ECHO telementoring. American Journal of Infection Control.

[CR34] Kidholm K, Jensen LK, Kjølhede T, Nielsen E, Horup MB (2018). Validity of the model for assessment of telemedicine: A Delphi study. Journal of Telemedicine and Telecare.

[CR35] Lewiecki EM, Boyle JF, Arora S, Bouchonville MF, Chafey DH (2017). Telementoring: A novel approach to reducing the osteoporosis treatment gap. Osteoporosis International.

[CR36] Linstone HA, Turoff M (1975). The Delphi method: Techniques and applications.

[CR37] McPhillips AM, Schultz RJ, Nasuta M, Shafer PO (2021). ECHO telementoring applied to managing students with seizures: The benefits for school nurses. Nasnewsletter.

[CR38] Moss P, Hartley N, Russell T (2021). Integration Intrapreneurship: Implementing Innovation in a Public Healthcare Organisation. Journal of Innovation and Entrepreneurship..

[CR39] Moss P, Hartley N, Ziviani J, Newcomb D, Russell T (2020). Executive decision-making: Piloting project ECHO to integrate care in Queensland. international Journal of Integrated Care.

[CR40] Nhung LH, Dien TM, Lan NP, Thanh PQ, Cuong PV (2021). Use of Project ECHO Telementoring Model in Continuing Medical Education for Pediatricians in Vietnam: Preliminary Results..

[CR41] Okoli C, Pawlowski SD (2004). The Delphi method as a research tool: An example, design considerations and applications. Information & Management.

[CR42] Schmidt RC (1997). Managing Delphi surveys using nonparametric statistical techniques. Decision Sciences.

[CR43] Serhal E, Arena A, Sockalingam S, Mohri L, Crawford A (2018). Adapting the consolidated framework for implementation research to create organizational readiness and implementation tools for project ECHO. Journal of Continuing Education in the Health Professions.

[CR44] Silva HP, Lehoux P, Hagemeister N (2018). Developing a tool to assess responsibility in health innovation: Results from an international Delphi study. Health Policy and Technology.

[CR45] Singh S, Sharma V, Patel P, Anuragi G, Sharma RG (2016). Telementoring: An overview and our preliminary experience in the setting up of a cost-effective telementoring facility. The Indian Journal of Surgery.

[CR46] Snyder-Halpern R (2001). Indicators of organizational readiness for clinical information technology/systems innovation: A Delphi study. International Journal of Medical Informatics.

[CR47] Socolovsky C, Masi C, Hamlish T, Aduana G, Arora S, Bakris G, Johnson D (2013). Evaluating the role of key learning theories in ECHO: A telehealth educational program for primary care providers. Progress in Community Health Partnerships.

[CR48] Tosi LL, Rajah EN, Stewart MH, Gillies AP, Hart TS, Lewiecki EM (2020). The rare bone disease TeleECHO program: Leveraging telehealth to improve rare bone disease care. Current Osteoporosis Reports.

[CR49] Wenger E, McDermott R, Snyder W (2002). Cultivating communities of practice: A guide to managing knowledge.

[CR50] Wild C, Torgersen H (2000). Foresight in medicine: Lessons from three European Delphi studies. European Journal of Public Health.

[CR51] Zhou C, Crawford A, Serhal E, Kurdyak P, Sockalingam S (2016). The impact of project ECHO on participant and patient outcomes: A systematic review. Academic Medicine.

