# Supplementary material for: Measuring the Success of a Project ECHO Implementation: Results from an International e-Delphi Study
Source: Glob Implement Res Appl. 2022 Aug 10;2(3):179–94. doi: 10.1007/s43477-022-00050-7 (PMC9365209; doi:10.1007/s43477-022-00050-7)
Supplement: Supplementary file 1 — Supplementary file1 (DOCX 58 kb) [file 43477_2022_50_MOESM1_ESM.docx]

**Supplementary Information**

**Appendices**

***Appendix 1:***

*Indicators of implementation success for Project ECHO – framework (N=54)*

*Note: The reference numbers for each indicator refers to the residual number of that indicator throughout the e-Delphi rounds. The reference numbers cited in Table 2 of the manuscript reflect the indicators grouped and numbered by domain.*

| # | Indicator of Success | Recommended Measurement Phase (Pre-Launch, Launch, Growth / Continuous Improvement) | Data Collection Point / Stakeholder (Individual Spoke Participant / Panellist, ECHO Network, ECHO Hub, Organisation, System) | Recommended method of Measurement |
| --- | --- | --- | --- | --- |
| Domain 1: Spoke Participant Engagement – 14 indicators  Definition: Indicators which measure the number, interactivity and participation experience of individuals who join ECHO Networks from a variety of spoke locations to connect and learn with panel teams centrally coordinated by the hub. | | | | |
| 1 | Spoke participants attend teleECHO Network sessions regularly. | Launch, Growth/ Continuous Improvement | Individual Spoke Participant | iECHO CRM teleECHO clinic attendance report: individual participant attendance. |
| 4 | Spoke participant diversity (gender, profession, culture, geography) attendance which meets target numbers. | Launch, Growth/ Continuous Improvement | Individual Spoke Participant | iECHO CRM teleECHO clinic attendance report: individual participant attendance. |
| 5 | Evidence of peer-to-peer testimonials. | Growth/ Continuous Improvement | Individual Spoke Participant | Documentation of/recordings of testimonials, Spoke Participant Surveys (individuals), Single Session Feedback (polling, surveys), Interviews and Focus Groups. |
| 11 | Higher levels of spoke participant experience (enjoyable, collegial, inclusive, non-judgemental). | Growth/ Continuous Improvement | Individual Spoke Participant | Spoke Participant Surveys (individuals), Single Session Feedback (polling, surveys), Interviews and Focus Groups. |
| 12 | Number of spoke participants who present cases for discussion. | Growth/ Continuous Improvement | Individual Spoke Participant | iECHO CRM participant report, iECHO CRM teleECHO clinic report. |
| 13 | Number of ECHO sessions where spoke participants present cases from their local context. | Growth/ Continuous Improvement | ECHO Network | iECHO CRM participant report, iECHO CRM teleECHO clinic report. |
| 14 | Higher levels of spoke participant safety and comfort in volunteering to present cases from their own context as a learning opportunity within the teleECHO Network. | Growth/ Continuous Improvement | Individual Spoke Participant | Spoke Participant Surveys (individuals), Single Session Feedback (polling, surveys), Interviews and Focus Groups, iECHO CRM teleECHO clinic report. |
| 18 | Higher levels of spoke participant satisfaction with didactic content, panel expert(s) representation/hub team support. | Growth/ Continuous Improvement | Individual Spoke Participant | Spoke Participant Surveys (individuals), Single Session Feedback (polling, surveys), Interviews and Focus Groups, iECHO CRM teleECHO clinic report. |
| 24 | Higher levels of spoke participant satisfaction with learning/advice/support gained from case presentation and discussion (applies to individual case presenter, as well as other spoke participants learning from the case) and recommendations. | Growth/ Continuous Improvement | Individual Spoke Participant | Spoke Participant Surveys (individuals), Single Session Feedback (polling, surveys), Interviews and Focus Groups. |
| 25 | Number of spoke participants who represent cases. | Launch, Growth/ Continuous Improvement | Individual Spoke Participant | iECHO CRM participant report, iECHO CRM teleECHO clinic report. |
| 38 | Higher levels of spoke participant satisfaction with the opportunity to contribute to the dialogue, ask questions, make recommendations whether verbally or non-verbally. | Growth/ Continuous Improvement | Individual Spoke Participant | Spoke Participant Surveys (individuals), Single Session Feedback (polling, surveys), Interviews and Focus Groups. |
| 41 | High reported levels of spoke participants self-reporting that they feel safe, supported, and welcomed at teleECHO Network sessions. | Growth/ Continuous Improvement | Individual Spoke Participant, ECHO Network | Spoke Participant Surveys (individuals), Single Session Feedback (polling, surveys), Interviews and Focus Groups, iECHO CRM teleECHO clinic report. |
| 45 | Measurable increase in spoke participants who contribute to the discussion verbally or via chat. | Growth/ Continuous Improvement | ECHO Network | teleECHO Scorecard. |
| 53 | Evidence of spoke participants inviting colleagues to attend teleECHO Network sessions to co-present case presentations. | Growth/ Continuous Improvement | Individual Spoke Participant, ECHO Network | iECHO CRM participant report, iECHO CRM teleECHO clinic report. |
| Domain 2: ECHO Hub / teleECHO Network design and operation – 23 indicators  Definition: Indicators which measure the design and operation of an organisation’s ECHO Hub, and / or individual ECHO Networks. | | | | |
| 15 | Evidence of the teleECHO Network's co-design occurred with prospective participants, consumers, system managers, and subject matter experts. | Pre-Launch, Growth/ Continuous Improvement | ECHO Network | Review of Implementation plan, Evaluation plan, Learning Needs Assessment results. |
| 16 | Number of discrete stakeholders involved in the co-design of the teleECHO Network. | Pre-Launch, Growth/ Continuous Improvement | ECHO Network | Review of Implementation plan, Evaluation plan, Learning Needs Assessment results. |
| 17 | Demonstrated alignment to local, state, federal priorities, and associated quality/funding metrics. | Pre-Launch, Growth/ Continuous Improvement | ECHO Network, ECHO Hub, Organisation, System | Review of Implementation plan, Evaluation plan, Learning Needs Assessment results, and teleECHO Network funding sources. |
| 19 | Evidence that the teleECHO Network delivers on the findings of the learning needs assessment. | Growth/ Continuous Improvement | ECHO Network, ECHO Hub. | Review of Implementation plan, Evaluation plan, Learning Needs Assessment results, Spoke Participant Surveys (individuals), Single Session Feedback (polling, surveys), Interviews and Focus Groups. |
| 37 | Measurable increase in levels of interactivity amongst spoke participants and panellists during sessions (on camera, chat, verbal, non-verbal, volunteering to present cases). | Growth/ Continuous Improvement | ECHO Network - Spoke Participants, Panellists | teleECHO Scorecard. |
| 43 | Frequency/ regularity of sessions - sessions are held routinely. | Launch, Growth/ Continuous Improvement | ECHO Network | iECHO CRM participant report, iECHO CRM teleECHO clinic report. |
| 44 | Higher levels of balance in dialogue contributed by panellists vs spoke participants, demonstrating spokes are contributing at least 50% of the talking. | Growth/ Continuous Improvement | ECHO Network | teleECHO Scorecard. |
| 48 | Number of teleECHO sessions including a participant case. | Launch, Growth/ Continuous Improvement | ECHO Network | iECHO CRM participant report, iECHO CRM teleECHO clinic report. |
| 49 | High levels of teleECHO sessions being a non-hierarchical, professional forum for knowledge sharing is fostered by panellists. | Launch, Growth/ Continuous Improvement | Individual ECHO Network Panellist, ECHO Network | teleECHO Scorecard, Spoke Participant Surveys (individuals), Single Session Feedback (polling, surveys), Interviews and Focus Groups. |
| 54 | Evidence of streamlined hub operational and logistical processes that optimise the delivery of teleECHO sessions. | Pre-Launch, Growth/ Continuous Improvement | ECHO Network, ECHO Hub | teleECHO Scorecard, iECHO CRM participant report, iECHO CRM teleECHO clinic report, evidence of localised policies, procedures, manuals for ECHO hub operations. |
| 60 | Evidence that the teleECHO Panel adheres to the Anatomy of an ECHO for fidelity assurance. | Launch, Growth/ Continuous Improvement | Individual ECHO Network Panellist, ECHO Network | teleECHO Scorecard, Spoke Participant Surveys (individuals), Single Session Feedback (polling, surveys), Interviews and Focus Groups. |
| 64 | Evidence of ECHO hub teams undertaking learner needs assessment, implementation planning, evaluation planning, panel expertise onboarding, and 2 mock ECHO sessions prior to launching a teleECHO Network. | Pre-Launch. | Individual ECHO Network Panellist, ECHO Hub | Review of Implementation plan, Evaluation plan, Learning Needs Assessment results, Interviews and Focus Groups, mock and post-launch teleECHO Scorecards. |
| 67 | Evidence of hub team attracting sufficient funding to fulfil implementation/ hub management/ replication functions sustainably. | Pre-Launch, Launch, Growth/ Continuous Improvement. | ECHO Hub | Organisational cost centre/financial reports. |
| 68 | Evidence of hub team managing operations within budget constraints of the organisation. | Launch, Growth/ Continuous Improvement | ECHO Hub | Organisational cost centre/financial reports. |
| 69 | Evidence of ECHO hub leadership role(s) and clear organisational governance oversight of ECHO hub team structure are present. | Pre-Launch, Launch, Growth/ Continuous Improvement. | ECHO Hub | ECHO team role descriptions, organisational structure, organisation's operational and/or strategic plans. |
| 70 | Evidence of an interprofessional and diverse hub team. | Pre-Launch, Launch, Growth/ Continuous Improvement. | ECHO Hub | ECHO team role descriptions, organisational structure. |
| 74 | Evidence of communication systems/processes developed for routine engagement with stakeholders outside of teleECHO sessions. | Launch, Growth/ Continuous Improvement | Individual ECHO Network Panellist, ECHO Hub | Correspondence records, mailing lists, templates/CRM for distribution of didactic resources, reference lists, journal articles, podcasts, and other resources. |
| 75 | Evidence of hub team’s development and dissemination of marketing materials to increase awareness of and attraction to their ECHO operations. | Pre-Launch, Launch, Growth/ Continuous Improvement. | Individual ECHO Network Panellist, ECHO Hub | Media analytics dashboards that can be tailored/shared widely across multiple stakeholder audiences as appropriate including content attesting to the quality/credibility of organisational hub team/panellists. Examples would include succinct and engaging marketing materials to increase awareness of and attraction to ECHO activities and be tailored/shared widely across multiple audiences. |
| 76 | Evidence of panellists and spoke participants advocating via word of mouth, peer-to-peer, personal/ professional network communication/ recommendations about joining teleECHO network(s). | Pre-Launch, Launch, Growth/ Continuous Improvement. | Individual Spoke Participant, Individual ECHO Network Panellist, ECHO Network | Qualitative documentation of/recordings of testimonials, Spoke Participant Surveys (individuals), Single Session Feedback (polling, surveys), Interviews and Focus Groups. |
| 78 | Evidence of hub stakeholders (champion, facilitator, panellists, coordinator) completing ECHO Immersion training provided by a designated Superhub prior to launch. | Pre-Launch. | Individual ECHO Network Panellist, ECHO Hub | Immersion attendance records. |
| 79 | Evidence of ECHO hub team engaging with Superhub for post-Immersion partner liaison support and mentorship. | Pre-Launch, Launch, Growth/ Continuous Improvement. | Individual ECHO Network Panellist, ECHO Hub | iECHO/Salesforce CRM reports (partner liaison, technical assistance). |
| 82 | Evidence of hub teams having data collection processes to ensure all pertinent data is collected and evaluated in a reliable way. | Pre-Launch, Launch, Growth/ Continuous Improvement. | ECHO Network Panel, ECHO Hub | teleECHO Scorecard records, iECHO CRM reports, evidence of localised protocols for ECHO hub data collection and evaluation. |
| 103 | Evidence of executive/ leadership support - where ECHO activities strategically align to organisational priorities, funding/investment decision-making. | Pre-Launch, Launch, Growth/ Continuous Improvement. | ECHO Hub, Organisation | Qualitative and quantitative documentation of/recordings of executive/leadership endorsement/advocacy for ECHO, reference points in organisational strategies, policies, plans, financial cost centre reports. |
| Domain 3: ECHO Hub team engagement – 5 indicators  Definition: Indicators which measure the number, interactivity and participation experience of individuals who facilitate and manage ECHO Hub functions. | | | | |
| 46 | High levels of panellist experience and satisfaction (enjoyable, high value, time efficient). | Growth/ Continuous Improvement | Individual ECHO Panellist, ECHO Network | Panellist Surveys (individuals), Single Session Feedback (polling, surveys), Interviews and Focus Groups. |
| 50 | Evidence of relevant stakeholders having a clear understanding of the ECHO model, its theoretical and practical application, and potential benefits. | Launch, Growth/ Continuous Improvement | Individual ECHO Network Panellist, ECHO Hub, Organisation | Review of Implementation plan, Evaluation plan, Learning Needs Assessment results, Interviews and Focus Groups. |
| 55 | High levels of strong and organised facilitation role/function, panel cohesion and satisfaction during teleECHO Network sessions. | Launch, Growth/ Continuous Improvement | Individual ECHO Network Panellist, ECHO Network | teleECHO Scorecard, evidence of localised policies, procedures, manuals for ECHO hub operations. |
| 56 | Demonstrated ability to recruit and retain Champion, Facilitator, Panellists with the right qualification, skills, expertise, lived experience, ability to present well and make spoke participants feel comfortable. | Launch, Growth/ Continuous Improvement | Individual ECHO Network Panellist, ECHO Network | teleECHO Scorecard, ECHO team role descriptions. |
| 57 | High levels of panel facilitator and panellist satisfaction with learning/ advice/ support contributed to/ arising from case presentation/s (applies to panel contributions, individual case presenter, as well as other spoke participants' learning and contribution to recommendations for the case/s). | Launch, Growth/ Continuous Improvement | Individual ECHO Network Panellist, ECHO Network | Panellist Surveys (individuals), Single Session Feedback (polling, surveys), Interviews and Focus Groups. |
| Domain 4: Local Impact – 12 indicators  Definition: Indicators which measure the increase or improvement in workforce development, capacity, system integration and efficiency. | | | | |
| 27 | Measurable increase in spoke participant's confidence to manage cases locally. | Pre-Launch, Growth/ Continuous Improvement | Individual Spoke Participant | Spoke Participant Surveys (individuals), Single Session Feedback (polling, surveys), Interviews and Focus Groups. |
| 28 | Measurable increase in spoke participant's competence to manage cases locally. | Pre-Launch, Growth/ Continuous Improvement | Individual Spoke Participant | Spoke Participant Surveys (individuals), Single Session Feedback (polling, surveys), Interviews and Focus Groups. |
| 29 | Measurable increase in spoke participant's knowledge/skills to manage cases locally. | Pre-Launch, Growth/ Continuous Improvement | Individual Spoke Participant | Spoke Participant Surveys (individuals), Single Session Feedback (polling, surveys), Interviews and Focus Groups. |
| 30 | Measurable increase in spoke participant's capacity to manage cases locally. | Pre-Launch, Growth/ Continuous Improvement | Individual Spoke Participant | Spoke Participant Surveys (individuals), Single Session Feedback (polling, surveys), Interviews and Focus Groups. |
| 31 | Measurable increase in spoke participant self-reported change in experience to become a local expert to whom colleagues in their community/proximity refer to and collaborate with for support on cases. | Pre-Launch, Growth/ Continuous Improvement | Individual Spoke Participant | Spoke Participant Surveys (individuals), Single Session Feedback (polling, surveys), Interviews and Focus Groups. |
| 32 | Spoke participants applying of at least one change in their practice due to their participation in teleECHO Networks. | Growth/ Continuous Improvement | Individual Spoke Participant | Spoke Participant Surveys (individuals), Single Session Feedback (polling, surveys), Interviews and Focus Groups. |
| 33 | Measurable increase in spoke participant self-efficacy. | Pre-Launch, Growth/ Continuous Improvement | Individual Spoke Participant | Spoke Participant Surveys (individuals), Single Session Feedback (polling, surveys), Interviews and Focus Groups. |
| 34 | Measurable reduction in spoke participant's sense of professional isolation. | Pre-Launch, Growth/ Continuous Improvement | Individual Spoke Participant | Spoke Participant Surveys (individuals), Single Session Feedback (polling, surveys), Interviews and Focus Groups. |
| 35 | Measurable increase in spoke participant's joy of work. | Pre-Launch, Growth/ Continuous Improvement | Individual Spoke Participant | Spoke Participant Surveys (individuals), Single Session Feedback (polling, surveys), Interviews and Focus Groups. |
| 52 | Higher spoke participant reported positive changes in knowledge-sharing relationships between colleagues locally. | Growth/ Continuous Improvement | Individual Spoke Participant, ECHO Network | Spoke Participant Surveys (individuals), Single Session Feedback (polling, surveys), Interviews and Focus Groups. |
| 97 | Improvements in service utilisation, service wait times, distance travelled to access services by patients/consumers/ clients. | Launch, Growth/ Continuous Improvement | Individual consumer, Individual ECHO Spoke Participant, ECHO Network, ECHO Hub, Organisation, System | teleECHO case presentation and patient/client record audits, postcode mapping, economic modelling, and analysis. |
| 98 | Improvements in spoke participant’s professional relationships, access to specialist services, referral pathways, informed decision-making, peer-to-peer supports outside of teleECHO sessions which impact their patient/client care/service provision/professional isolation. | Launch, Growth/ Continuous Improvement | Individual Spoke Participant | Qualitative documentation of/recordings of testimonials, Spoke Participant Surveys (individuals), Single Session Feedback (polling, surveys), Interviews and Focus Groups. Social Network Analyses. |

***Appendix 2:***

*List of verified indicators after Round 2 (N=121)*

| # | Indicator of Success |
| --- | --- |
| 1 | Regular spoke participant attendance at teleECHO Network sessions. |
| 2 | Number of spoke participants. |
| 3 | Number of spoke participants attending more than 1 session, as well as those who attend sporadically/regularly throughout each series. |
| 4 | Target/priority/interprofessional/urban, regional, rural/diverse participants registering to attend as spokes, with attrition managed. |
| 5 | Peer to peer testimonials. |
| 6 | Using professional development credits to incentivise spoke participant registration and attendance. Useful to look at uptake rates of credits. |
| 7 | Spoke participant diversity (gender, profession, culture). |
| 8 | Range of spoke participant attendance (many spokes attending, new spokes joining vs same 2-3 spoke participants per session). |
| 9 | Spoke participant attendance is at approximately 75% for all sessions for each participant. |
| 10 | Percentage of registered spoke participants who attend each session. |
| 11 | Percentage of spoke participants who submit cases. |
| 12 | Measurable spoke participant experience (enjoyable, collegial, inclusive, non-judgemental). |
| 13 | Number of spoke participants who present cases for discussion. |
| 14 | Spoke participants regularly provide suitable cases from their local context for presentation in teleECHO sessions. |
| 15 | There is a sense of safety and comfort in spoke participants volunteering to present cases from their own context as a learning opportunity with the teleECHO Network. |
| 16 | Spoke participants provide a minimum of two new cases per session for presentation and discussion. |
| 17 | TeleECHO Network is co-designed with prospective spoke participants, patients/consumers, system managers, subject matter experts. |
| 18 | Co-design is deliberate with potential spoke participants and patients/consumers to ensure the ECHO concept has motivated buy-in and that end consumer/beneficiary needs are considered. |
| 19 | Alignment where possible to local, state, federal priorities, and associated quality/funding metrics. |
| 20 | Measurable spoke participant satisfaction with didactic content, panel expert(s) representation/hub team support. |
| 21 | Relevance of the teleECHO network's didactic content and expertise of panellists to the spoke participant's local practice/role/location is clearly attributable to a learning needs assessment. |
| 22 | Spoke participant self-report the ECHO network's focus area is relevant to their personal/professional interest at the point of initial registration to join the network. |
| 23 | Spoke participants confirm that ECHO Network topics respond to demonstrated interests that spoke participants identified during learning needs assessments, leading to consistent participant recruitment, and high engagement/attendance in sessions. |
| 24 | Measurable learnings from each case presentation for each participant in teleECHO sessions, regardless of their profession/role/location. |
| 25 | Relevance of the case presentation and recommendations content to the wider spoke participants' local practice/role/location is clear. |
| 26 | Measurable spoke participant satisfaction with learning/advice/support gained from case presentation and discussion (applies to individual case presenter, as well as other spoke participants learning from the case) and recommendations that are co-designed by all parties. |
| 27 | Number and growth of spoke participants who represent cases that demonstrate change in management. |
| 28 | Ongoing dialogue/follow-up with case presenters to understand application and outcomes following the actioning of any case recommendations, or more advice required through a subsequent in-session case presentation. |
| 29 | Measurable increase in spoke participant's confidence to manage cases locally. |
| 30 | Measurable increase in spoke participant's competence to manage cases locally. |
| 31 | Measurable increase in spoke participant's knowledge/skills to manage cases locally. |
| 32 | Measurable increase in spoke participant's capacity to manage cases locally. |
| 33 | Spoke participants becoming local experts to whom colleagues in their community/proximity refer to and collaborate with for support on cases. |
| 34 | Spoke participants applying of a number of changes in their practice immediately following participation in ECHO sessions. |
| 35 | Measurable increase in spoke participant self-efficacy. |
| 36 | Measurable reduction in spoke participant's sense of professional isolation. |
| 37 | Measurable increase in spoke participant's joy of work. |
| 38 | Pre-determined case presentation schedule for spoke participants to ensure that everyone gets a chance to share a case from their local context for advice and support. |
| 39 | Regular spoke participants from one teleECHO Network cohort repeat attendance at subsequent cycle, and/or go on to participate in other teleECHO Networks. |
| 40 | Multi-site evidence of teleECHO networks being fit for purpose to target specific focus areas (Hepatitis, Autism, Mental Health & Behaviour, Diabetes, Education, Opioids, COVID-19, Quality Improvement), spoke participant demographics (health, education, child safety, defence, laboratory staff, consumers), demand/interest (frequency of sessions, registration rates), etc in multiple jurisdictions (local, state, national, international). |
| 41 | Measurable change in levels of interactivity amongst spoke participants and panellists during sessions (on camera, chat, verbal, non-verbal, volunteering to present cases). |
| 42 | Spoke participant satisfaction with the opportunity to contribute to the dialogue, ask questions, make recommendations whether verbally or non-verbally. |
| 43 | Interactivity helps to build community of practice that allows for vulnerability and learning. |
| 44 | Spoke participant attendance is regular to build rapport with other participants, such that a community of practice forms/grows. |
| 45 | Participants are made to feel safe, supported and welcomed. |
| 46 | Optimal size of spoke participant numbers in teleECHO sessions may vary but such that interactivity is not impaired. |
| 47 | Frequency/regularity of sessions - sessions are held to maximise the opportunity for "low-dose, high-frequency" ideal ECHO design for adult learners. |
| 48 | Measurable balance of dialogue contributed by panellists vs spoke participants to ensure spokes are contributing at least 50% of the talking. |
| 49 | Percentage of spoke participants who contribute to the discussion verbally or via chat. |
| 50 | Measurable change in panellist experience (enjoyable, high value, time efficient). |
| 51 | Measurable adherence to the Anatomy of an ECHO for fidelity assurance, and hub operations align to the values and ethos of the ECHO model. |
| 52 | TeleECHO sessions included a participant case, as an indication of: commitment to the Community of Practice and specifically, its practice development. |
| 53 | A non-hierarchical, professional forum for knowledge sharing is fostered by panellists. |
| 54 | Relevant stakeholders have a clear understanding of the ECHO model, its theoretical and practical application, and potential benefits. |
| 55 | Measurable change in how spoke participants interact with panel experts and/or colleagues outside of teleECHO sessions. |
| 56 | Measurable increase in spoke participants' sense of safety and support provided by membership in the Community of Practice. |
| 57 | Participant reports of changes in knowledge-sharing relationships between individuals (clinicians, educators, and social care providers) through a retrospective pre-/post self-assessment questionnaire. E.g., collaboration and advice-seeking behaviours with new discipline groups, locally. |
| 58 | Spoke participants invite colleagues from local team (internal/external) to attend teleECHO Network to co-present case presentation. Fosters interprofessional practice, shared care/management of cases. |
| 59 | Infrastructure, equipment, software, coordination functions (registration/enrolment portal, diary management, email correspondence, session agenda, internet bandwidth, scheduling) optimises the spoke participants and hub team's optimal ability to participate and interact in teleECHO sessions. |
| 60 | Strong and organised facilitation role/function, panel cohesion and satisfaction during teleECHO Network sessions. |
| 61 | Champion, Facilitator, Panellists: need to recruit and retain people with the right qualification, skills, expertise, lived experience, ability to present well and make spoke participants feel comfortable. |
| 62 | Measurable panel facilitator and member satisfaction with learning/advice/support contributed to/arising from case presentation/s (applies to panel contribution(s), individual case presenter, as well as other spoke participants' learning and contribution to recommendations for the case/s). |
| 63 | Measurable panel member satisfaction with experience of personal/professional development as a result of their ECHO roles. |
| 64 | Panel subject matter experts include staff, colleagues/external partners, consumer representatives. |
| 65 | Panel operates as cohesive and functional team that adheres to Anatomy of an ECHO fidelity, organisational/ECHO mission/values, and practices pre/debriefing to optimise in-session interactivity. |
| 66 | Coordinators have a complimentary background to topic area, authenticity and interest in the network topic, connection to already existing networks and personable. Coordinators function as conduit between the panel and participants. |
| 67 | IT support resource: access to internal IT support/troubleshooting skills/support as required. |
| 68 | ECHO panel team composition includes requisite subject matter expertise and professional representation, avoiding duplication of professional representatives. Panel representation can be sustained by the hub organisation ongoing. |
| 69 | Hub teams undertake learner needs assessment to inform curricula development, implementation planning, evaluation planning, panel expertise onboarding, 2 mock ECHO sessions. |
| 70 | Hub teams pilot sessions to identify gaps, stakeholder engagement, participant recruitment and onboarding, and subsequently launch a teleECHO network on schedule. |
| 71 | Hubs launching teleECHO networks with at least 10 spoke participants registered. |
| 72 | ECHO hub launches a teleECHO network. |
| 73 | Hub team has project management experience. |
| 74 | Hub team attracts sufficient/growth funding to fulfil implementation/hub management/replication functions sustainably. |
| 75 | Hub team manages operations within budget constraints of the organisation. |
| 76 | Hub Leadership role(s) and clear organisational governance oversight of ECHO hub team structure to be present. |
| 77 | Interprofessional and diverse hub team where everyone knows their panel role and contributes to fidelity to the ECHO model across all aspects of the hub operations. |
| 78 | Hub team includes representation from the target spoke participant audience/sector (can include part-time, in-kind, ex-officio, guest panellist roles). |
| 79 | Dedicated ECHO human resourcing/quarantined time for part-time role functions to allow for sufficient internal capacity amongst team to undertake hub management/replication/ panel functions within their workload. |
| 80 | Hub teams have a centralised administrative function within their organisation to ensure the consistent management of all ECHO-related functions (website, recruitment, communications, professional development credits, etc). |
| 81 | Communication systems/processes are developed for routine engagement with stakeholders outside of teleECHO sessions - including distributing didactic resources, reference lists, journal articles, podcasts, and other resources. |
| 82 | Hub teams develop and disseminate succinct, engaging marketing materials to increase awareness of and attraction to their ECHO operations, that can be tailored/shared widely across multiple stakeholder audiences as appropriate including content attesting to the quality/credibility of organisational hub team/panellists. |
| 83 | Panellists and spoke participants advocate via word of mouth, peer-to-peer, personal/professional network communication/recommendations about joining teleECHO network(s). |
| 84 | Hub teams have access to marketing and public relations support to create/develop promotional materials or social media content to translate and convey the mission/vision into something that draws attention. |
| 85 | Evidence of communication and positive engagement between hub team and leadership roles at spoke sites outside of ECHO sessions. |
| 86 | Hub, panel, other relevant staff complete ECHO Immersion training provided by a designated Superhub. |
| 87 | Accessibility to Immersion training provider, partner liaison support and mentorship in replication and growth phases. |
| 88 | Access to MetaECHO community resources (including PERL) to inform and support ECHO implementation and replication activities. |
| 89 | Individual ECHO hubs launching additional teleECHO networks to expand hub operations. |
| 90 | Routine data entry - including in iECHO CRM platform to track registration, demographics, attendance, case presentations in teleECHO sessions. |
| 91 | Hub teams have data collection processes to ensure all pertinent data is collected and evaluated in a reliable way. |
| 92 | Measurement of how well the proposed implementation addresses the identified problem(s), takes advantage of the opportunities identified by using the ECHO model, how it addresses the learning and cultural objectives of the spoke participants and principles of adult learning during the learning needs assessment(s)/planning phase. |
| 93 | Measurement of how the teleECHO network's goals/impact/achievement continues to attract investment to sustain the virtual Community of Practice. |
| 94 | Routine collection and reporting of qualitative and quantitative feedback following each single session/cohort cycle of teleECHO Networks to inform impact, quality improvement processes and innovation. |
| 95 | Measurable panel and spoke participant satisfaction with ECHO model as preferred mode of delivery: All stakeholders indicate satisfaction with the ECHO model as the preferred/optimal mode of delivery, as opposed to alternatives: face-to-face in-services, webinars, grand rounds, resource guides, lectures, etc. |
| 96 | Measurable change in panel member's time investment in knowledge sharing, mentorship, in-depth handovers pre/post ECHO. |
| 97 | Organisational objective/area of focus is a suitable fit for delivery by an ECHO Network approach (either: cohorts for CPD/CME; drop-ins; or a continuous/Community of Practice format). |
| 98 | Hub teams undertake routine focus groups, interviews with target stakeholder groups (panellists, spoke participants, other) at timepoints (pre- and post-launch) to evaluate if the implementation was successful. |
| 99 | Hub teams develop and implement a relevant and clearly stated vision and mission to guide the work of the ECHO Network. |
| 100 | ECHO Hub teams indicate satisfaction that the ECHO model is valuable in a significantly different way to alternatives. |
| 101 | Measurable change in population outcomes associated with ECHO spoke participation, case presentation, recommendations, continuity of care/service. |
| 102 | Measurable change in patient/other population outcomes being managed more effectively, as attributed to support provided through teleECHO sessions. |
| 103 | Measurable change in rates of screening, detection, diagnoses, prescribing, referral/caseload management at a population level attributed to support provided through teleECHO sessions. |
| 104 | Measurable change in low-value care/service provision at a population level. |
| 105 | Measurable change in costs to the consumer/beneficiary. |
| 106 | Spoke participant identifying a number of actual reported changes in their practice or thinking as a result in participation in ECHO sessions. |
| 107 | Measurable change in service utilisation, service wait times, distance travelled to access services by patients/consumers/clients. |
| 108 | Measurable change in spoke participants' professional relationships, access to specialist services, referral pathways, informed decision-making, peer-to-peer supports outside of teleECHO sessions which impact their patient/client care/service provision/professional isolation: this is broader than distinct number of patient/consumer beneficiaries attributable to case presentations, this refers to the wider diaspora of benefits associated from one unique spoke participant's involvement in a teleECHO network. |
| 109 | Measurable change in systems integration attributable to teleECHO Network(s). |
| 110 | Measurable change in system capacity/efficiency/quality to provide services/care attributable to teleECHO Network(s). |
| 111 | Measurable change in costs to the system/service provider(s) - including reduction/prevention of low-value care/service provision attributable to teleECHO Network(s). |
| 112 | Hub teams undertake a Most Significant Change (MSC) evaluation for their community of practice for participatory monitoring and evaluation. |
| 113 | Data collected by the ECHO hub team is used to establish a publication/presentation track record highlighting their contribution to the ECHO evidence base. |
| 114 | Executive/leadership support - where ECHO activities strategically align to organisational priorities, funding/investment decision-making. |
| 115 | Support, collaboration and buy-in from non-executive, non-leadership staff within the organisation who are not directly involved in ECHO activities. This leads to access to new/ongoing internal expert resources, growth/diversity in internal organisational interest. |
| 116 | Creation of an internal application process/pipeline to launch new additional teleECHO networks by other internal staff of hub organisations. |
| 117 | System/Sectoral uptake of the ECHO model as a preferred solution to address goals/challenges. |
| 118 | ECHO hubs partner with, are invested in by funders, insurers, research, philanthropic organisations to sustain, innovate, expand, evaluate ECHO activities, to amplify impact. |
| 119 | Measurable growth and adoption of the ECHO model by regional/rural as well as metropolitan stakeholders/organisations implementing and establishing ECHO hub operations. Distinctly different from adoption by spoke participants. |
| 120 | ECHO becomes normalised within the service delivery context of any sector in which it is used. |
| 121 | Measurable change in hub team's interaction with peers at other hubs to learn, share and collaborate on efforts using the ECHO model for various goals and objectives (area of focus, quality improvement, research, grants, etc). |

***Appendix 3:***

*List of indicators not retained after Round 3 (N=12)*

| # | Indicator of Success |
| --- | --- |
| 9 | Spoke participant attendance is at approximately 75% for all sessions for each participant. |
| 16 | Spoke participants provide a minimum of two new cases per session for presentation and discussion. |
| 38 | Pre-determined case presentation schedule for spoke participants to ensure that everyone gets a chance to share a case from their local context for advice and support. |
| 39 | Regular spoke participants from one teleECHO Network cohort repeat attendance at subsequent cycle, and/or go on to participate in other teleECHO Networks. |
| 55 | Measurable change in how spoke participants interact with panel experts and/or colleagues outside of teleECHO sessions. |
| 71 | Hubs launching teleECHO networks with at least 10 spoke participants registered. |
| 73 | Hub team has project management experience. |
| 84 | Hub teams have access to marketing and public relations support to create/develop promotional materials or social media content to translate and convey the mission/vision into something that draws attention. |
| 89 | Individual ECHO hubs launching additional teleECHO networks to expand hub operations. |
| 96 | Measurable change in panel member's time investment in knowledge sharing, mentorship, in-depth handovers pre/post ECHO. |
| 112 | Hub teams undertake a Most Significant Change (MSC) evaluation for their community of practice for participatory monitoring and evaluation. |
| 116 | Creation of an internal application process/pipeline to launch new additional teleECHO networks by other internal staff of hub organisations. |

***Appendix 4:***

*List of indicators not retained after Round 4 (N=37)*

| # | Indicator of Success |
| --- | --- |
| 2 | Number of spoke participants. |
| 3 | Number of spoke participants attending more than 1 session, as well as those who attend sporadically/regularly throughout each series. |
| 6 | Using professional development credits to incentivise spoke participant registration and attendance. Useful to look at uptake rates of credits. |
| 8 | Range of spoke participant attendance (many spokes attending, new spokes joining vs same 2-3 spoke participants per session). |
| 9 | Percentage of registered spoke participants who attend each session. |
| 10 | Percentage of spoke participants who submit cases. |
| 22 | Measurable learnings from each case presentation for each participant in teleECHO sessions, regardless of their profession/role/location. |
| 26 | Ongoing dialogue/follow-up with case presenters to understand application and outcomes following the actioning of any case recommendations, or more advice required through a subsequent in-session case presentation. |
| 36 | Multi-site evidence of teleECHO networks being fit for purpose to target specific focus areas (Hepatitis, Autism, Mental Health & Behaviour, Diabetes, Education, Opioids, COVID-19, Quality Improvement), spoke participant demographics (health, education, child safety, defence, laboratory staff, consumers), demand/interest (frequency of sessions, registration rates), etc in multiple jurisdictions (local, state, national, international). |
| 61 | Coordinators have a complimentary background to topic area, authenticity and interest in the network topic, connection to already existing networks and personable. Coordinators function as conduit between the panel and participants. |
| 62 | IT support resource: access to internal IT support/troubleshooting skills/support as required. |
| 65 | Hub teams pilot sessions to identify gaps, stakeholder engagement, participant recruitment and onboarding, and subsequently launch a teleECHO network on schedule. |
| 66 | ECHO hub launches a teleECHO network. |
| 71 | Hub team includes representation from the target spoke participant audience/sector (can include part-time, in-kind, ex-officio, guest panellist roles). |
| 72 | Dedicated ECHO human resourcing/quarantined time for part-time role functions to allow for sufficient internal capacity amongst team to undertake hub management/replication/ panel functions within their workload. |
| 73 | Hub teams have a centralised administrative function within their organisation to ensure the consistent management of all ECHO-related functions (website, recruitment, communications, professional development credits, etc). |
| 77 | Evidence of communication and positive engagement between hub team and leadership roles at spoke sites outside of ECHO sessions. |
| 80 | Access to MetaECHO community resources (including PERL) to inform and support ECHO implementation and replication activities. |
| 84 | Measurement of how the teleECHO network's goals/impact/achievement continues to attract investment to sustain the virtual Community of Practice. |
| 85 | Routine collection and reporting of qualitative and quantitative feedback following each single session/cohort cycle of teleECHO Networks to inform impact, quality improvement processes and innovation. |
| 86 | Measurable panel and spoke participant satisfaction with ECHO model as preferred mode of delivery: All stakeholders indicate satisfaction with the ECHO model as the preferred/optimal mode of delivery, as opposed to alternatives: face-to-face in-services, webinars, grand rounds, resource guides, lectures, etc. |
| 87 | Organisational objective/area of focus is a suitable fit for delivery by an ECHO Network approach (either: cohorts for CPD/CME; drop-ins; or a continuous/Community of Practice format). |
| 88 | Hub teams undertake routine focus groups, interviews with target stakeholder groups (panellists, spoke participants, other) at timepoints (pre- and post-launch) to evaluate if the implementation was successful. |
| 89 | Hub teams develop and implement a relevant and clearly stated vision and mission to guide the work of the ECHO Network. |
| 91 | Measurable change in population outcomes associated with ECHO spoke participation, case presentation, recommendations, continuity of care/service. |
| 92 | Measurable change in patient/other population outcomes being managed more effectively, as attributed to support provided through teleECHO sessions. |
| 93 | Measurable change in rates of screening, detection, diagnoses, prescribing, referral/caseload management at a population level attributed to support provided through teleECHO sessions. |
| 94 | Measurable change in low-value care/service provision at a population level. |
| 95 | Measurable change in costs to the consumer/beneficiary. |
| 100 | Measurable change in system capacity/efficiency/quality to provide services/care attributable to teleECHO Network(s). |
| 101 | Measurable change in costs to the system/service provider(s) - including reduction/prevention of low-value care/service provision attributable to teleECHO Network(s). |
| 102 | Data collected by the ECHO hub team is used to establish a publication/presentation track record highlighting their contribution to the ECHO evidence base. |
| 104 | Support, collaboration and buy-in from non-executive, non-leadership staff within the organisation who are not directly involved in ECHO activities. This leads to access to new/ongoing internal expert resources, growth/diversity in internal organisational interest. |
| 105 | System/Sectoral uptake of the ECHO model as a preferred solution to address goals/challenges. |
| 106 | ECHO hubs partner with, are invested in by funders, insurers, research, philanthropic organisations to sustain, innovate, expand, evaluate ECHO activities, to amplify impact. |
| 107 | Measurable growth and adoption of the ECHO model by regional/rural as well as metropolitan stakeholders/organisations implementing and establishing ECHO hub operations. Distinctly different from adoption by spoke participants. |
| 109 | Measurable change in hub team's interaction with peers at other hubs to learn, share and collaborate on efforts using the ECHO model for various goals and objectives (area of focus, quality improvement, research, grants, etc). |

***Appendix 5:***

*Amalgamated Indicators (N=7)*

| # | Indicator of Success | Final Phase Revision Outcome |
| --- | --- | --- |
| 7 | Spoke participant diversity (gender, profession, culture). | Amalgamate with Item 4. |
| 47 | Measurable adherence to the Anatomy of an ECHO for fidelity assurance, and hub operations align to the values and ethos of the ECHO model. | Amalgamated with Item 60. |
| 51 | Measurable increase in spoke participants’ sense of safety and support provided by membership in the Community of Practice. | Amalgamated with Item 41. |
| 58 | Measurable panel member satisfaction with experience of personal/professional development as a result of their ECHO roles. | Amalgamated with Item 57. |
| 59 | Panel subject matter experts include staff, colleagues/external partners, consumer representatives. | Amalgamated with Item 19. |
| 63 | ECHO panel team composition includes requisite subject matter expertise and professional representation, avoiding duplication of professional representatives. Panel representation can be sustained by the hub organisation ongoing. | Amalgamated with Item 19. |
| 81 | Routine data entry - including in iECHO CRM platform to track registration, demographics, attendance, case presentations in teleECHO sessions. | Amalgamate with Item 82. |

***Appendix 6:***

*Indicators with minor word edits (N=46)*

| # | Indicator of Success | Final Phase Revision Outcome |
| --- | --- | --- |
| 1 | Regular spoke participant attendance at teleECHO Network sessions. | Spoke participants attend teleECHO Network sessions regularly. |
| 4 | Target/priority/interprofessional/urban, regional, rural/diverse participants registering to attend as spokes, with attrition managed. | Spoke participant diversity (gender, profession, culture, geography) attendance which meets target numbers. |
| 5 | Peer to peer testimonials. | Evidence of peer-to-peer testimonials. |
| 11 | Measurable spoke participant experience (enjoyable, collegial, inclusive, non-judgemental). | Higher levels of spoke participant experience (enjoyable, collegial, inclusive, non-judgemental). |
| 13 | Spoke participants regularly provide suitable cases from their local context for presentation in teleECHO sessions. | Number of ECHO sessions where spoke participants present cases from their local context. |
| 14 | There is a sense of safety and comfort in spoke participants volunteering to present cases from their own context as a learning opportunity with the teleECHO Network. | Higher levels of spoke participant safety and comfort in volunteering to present cases from their own context as a learning opportunity within the teleECHO Network. |
| 15 | TeleECHO Network is co-designed with prospective spoke participants, patients/consumers, system managers, subject matter experts. | Evidence of the teleECHO Network's co-design occurred with prospective participants, consumers, system managers, and subject matter experts. |
| 16 | Co-design is deliberate with potential spoke participants and patients/consumers to ensure the ECHO concept has motivated buy-in and that end consumer/beneficiary needs are considered. | Number of discrete stakeholders involved in the co-design of the teleECHO Network. |
| 17 | Alignment where possible to local, state, federal priorities, and associated quality/funding metrics. | Demonstrated alignment to local, state, federal priorities, and associated quality/funding metrics. |
| 18 | Measurable spoke participant satisfaction with didactic content, panel expert(s) representation/hub team support. | Higher levels of spoke participant satisfaction with didactic content, panel expert(s) representation/hub team support. |
| 19 | Relevance of the teleECHO network's didactic content and expertise of panellists to the spoke participant's local practice/role/location is clearly attributable to a learning needs assessment. | Evidence that the teleECHO Network delivers on the findings of the learning needs assessment. |
| 24 | Measurable spoke participant satisfaction with learning/advice/support gained from case presentation and discussion (applies to individual case presenter, as well as other spoke participants learning from the case) and recommendations that are co-designed by all parties. | Higher levels of spoke participant satisfaction with learning/advice/support gained from case presentation and discussion (applies to individual case presenter, as well as other spoke participants learning from the case) and recommendations. |
| 25 | Number and growth of spoke participants who represent cases that demonstrate change in management. | Number of spoke participants who represent cases. |
| 31 | Spoke participants becoming local experts to whom colleagues in their community/proximity refer to and collaborate with for support on cases. | Measurable increase in spoke participant self-reported change in experience to become a local expert to whom colleagues in their community/proximity refer to and collaborate with for support on cases. |
| 32 | Spoke participants applying of a number of changes in their practice immediately following participation in ECHO sessions. | Spoke participants applying of at least one change in their practice due to their participation in teleECHO Networks. |
| 37 | Measurable change in levels of interactivity amongst spoke participants and panellists during sessions (on camera, chat, verbal, non-verbal, volunteering to present cases). | Measurable increase in levels of interactivity amongst spoke participants and panellists during sessions (on camera, chat, verbal, non-verbal, volunteering to present cases). |
| 38 | Spoke participant satisfaction with the opportunity to contribute to the dialogue, ask questions, make recommendations whether verbally or non-verbally. | Higher levels of spoke participant satisfaction with the opportunity to contribute to the dialogue, ask questions, make recommendations whether verbally or non-verbally. |
| 41 | Participants are made to feel safe, supported and welcomed. | High reported levels of spoke participants self-reporting that they feel safe, supported, and welcomed at teleECHO Network sessions. |
| 43 | Frequency/regularity of sessions - sessions are held to maximise the opportunity for "low-dose, high-frequency" ideal ECHO design for adult learners. | Frequency/regularity of sessions - sessions are held routinely. |
| 44 | Measurable balance of dialogue contributed by panellists vs spoke participants to ensure spokes are contributing at least 50% of the talking. | Higher levels of balance in dialogue contributed by panellists vs spoke participants, demonstrating spokes are contributing at least 50% of the talking. |
| 45 | Percentage of spoke participants who contribute to the discussion verbally or via chat. | Measurable increase in spoke participants who contribute to the discussion verbally or via chat. |
| 46 | Measurable change in panellist experience (enjoyable, high value, time efficient). | High levels of panellist experience and satisfaction (enjoyable, high value, time efficient). |
| 48 | TeleECHO sessions included a participant case, as an indication of: commitment to the Community of Practice and specifically, its practice development. | Number of teleECHO sessions including a participant case. |
| 49 | A non-hierarchical, professional forum for knowledge sharing is fostered by panellists. | High levels of teleECHO sessions being a non-hierarchical, professional forum for knowledge sharing is fostered by panellists. |
| 50 | Relevant stakeholders have a clear understanding of the ECHO model, its theoretical and practical application, and potential benefits. | Evidence of relevant stakeholders having a clear understanding of the ECHO model, its theoretical and practical application, and potential benefits. |
| 52 | Participant reports of changes in knowledge-sharing relationships between individuals (clinicians, educators, and social care providers) through a retrospective pre-/post self-assessment questionnaire. E.g., collaboration and advice-seeking behaviours with new discipline groups, locally. | Higher spoke participant reported positive changes in knowledge-sharing relationships between colleagues locally. |
| 53 | Spoke participants invite colleagues from local team (internal/external) to attend teleECHO Network to co-present case presentation. Fosters interprofessional practice, shared care/management of cases. | Evidence of spoke participants inviting colleagues to attend teleECHO Network sessions to co-present case presentations. |
| 54 | Infrastructure, equipment, software, coordination functions (registration/enrolment portal, diary management, email correspondence, session agenda, internet bandwidth, scheduling) optimises the spoke participants and hub team's optimal ability to participate and interact in teleECHO sessions. | Evidence of streamlined hub operational and logistical processes that optimise the delivery of teleECHO sessions. |
| 55 | Strong and organised facilitation role/function, panel cohesion and satisfaction during teleECHO Network sessions. | High levels of strong and organised facilitation role/function, panel cohesion and satisfaction during teleECHO Network sessions. |
| 56 | Champion, Facilitator, Panellists: need to recruit and retain people with the right qualification, skills, expertise, lived experience, ability to present well and make spoke participants feel comfortable. | Demonstrated ability to recruit and retain Champion, Facilitator, Panellists with the right qualification, skills, expertise, lived experience, ability to present well and make spoke participants feel comfortable. |
| 57 | Measurable panel facilitator and member satisfaction with learning/advice/support contributed to/arising from case presentation/s (applies to panel contributions, individual case presenter, as well as other spoke participants' learning and contribution to recommendations for the case/s). | High levels of panel facilitator and panellist satisfaction with learning/advice/support contributed to/arising from case presentation/s (applies to panel contributions, individual case presenter, as well as other spoke participants' learning and contribution to recommendations for the case/s). |
| 60 | Panel operates as cohesive and functional team that adheres to Anatomy of an ECHO fidelity, organisational/ECHO mission/values, and practices pre/debriefing to optimise in-session interactivity. | Evidence that the teleECHO Panel adheres to the Anatomy of an ECHO for fidelity assurance. |
| 64 | Hub teams undertake learner needs assessment to inform curricula development, implementation planning, evaluation planning, panel expertise onboarding, 2 mock ECHO sessions. | Evidence of ECHO hub teams undertaking learner needs assessment, implementation planning, evaluation planning, panel expertise onboarding, and 2 mock ECHO sessions prior to launching a teleECHO Network. |
| 67 | Hub team attracts sufficient/growth funding to fulfil implementation/hub management/replication functions sustainably. | Evidence of hub team attracting sufficient funding to fulfil implementation/hub management/replication functions sustainably. |
| 68 | Hub team manages operations within budget constraints of the organisation. | Evidence of hub team managing operations within budget constraints of the organisation. |
| 69 | Hub Leadership role(s) and clear organisational governance oversight of ECHO hub team structure to be present. | Evidence of ECHO hub leadership role(s) and clear organisational governance oversight of ECHO hub team structure are present. |
| 70 | Interprofessional and diverse hub team where everyone knows their panel role and contributes to fidelity to the ECHO model across all aspects of the hub operations. | Evidence of an interprofessional and diverse hub team. |
| 74 | Communication systems/processes are developed for routine engagement with stakeholders outside of teleECHO sessions - including distributing didactic resources, reference lists, journal articles, podcasts, and other resources. | Evidence of communication systems/processes developed for routine engagement with stakeholders outside of teleECHO sessions. |
| 75 | Hub teams develop and disseminate succinct, engaging marketing materials to increase awareness of and attraction to their ECHO operations, that can be tailored/shared widely across multiple stakeholder audiences as appropriate including content attesting to the quality/credibility of organisational hub team/panellists. | Evidence of hub team’s development and dissemination of marketing materials to increase awareness of and attraction to their ECHO operations. |
| 76 | Panellists and spoke participants advocate via word of mouth, peer-to-peer, personal/professional network communication/recommendations about joining teleECHO network(s). | Evidence of panellists and spoke participants advocating via word of mouth, peer-to-peer, personal/professional network communication/recommendations about joining teleECHO network(s). |
| 78 | Hub, panel, other relevant staff complete ECHO Immersion training provided by a designated Superhub. | Evidence of hub stakeholders (champion, facilitator, panellists, coordinator) completing ECHO Immersion training provided by a designated Superhub prior to launch. |
| 79 | Accessibility to Immersion training provider, partner liaison support and mentorship in replication and growth phases. | Evidence of ECHO hub team engaging with Superhub for post-Immersion partner liaison support and mentorship. |
| 82 | Hub teams have data collection processes to ensure all pertinent data is collected and evaluated in a reliable way. | Evidence of hub teams having data collection processes to ensure all pertinent data is collected and evaluated in a reliable way. |
| 97 | Measurable change in service utilisation, service wait times, distance travelled to access services by patients/consumers/clients. | Improvements in service utilisation, service wait times, distance travelled to access services by patients/consumers/clients. |
| 98 | Measurable change in spoke participants' professional relationships, access to specialist services, referral pathways, informed decision-making, peer-to-peer supports outside of teleECHO sessions which impact their patient/client care/service provision/professional isolation: this is broader than distinct number of patient/consumer beneficiaries attributable to case presentations, this refers to the wider diaspora of benefits associated from one unique spoke participant's involvement in a teleECHO network. | Improvements in spoke participants' professional relationships, access to specialist services, referral pathways, informed decision-making, peer-to-peer supports outside of teleECHO sessions which impact their patient/client care/service provision/professional isolation. |
| 103 | Executive/leadership support - where ECHO activities strategically align to organisational priorities, funding/investment decision-making. | Evidence of executive/leadership support - where ECHO activities strategically align to organisational priorities, funding/investment decision-making. |

***Appendix 7:***

*Redundant Indicators (N=11)*

| # | Indicator of Success | Final Phase Revision Outcome |
| --- | --- | --- |
| 20 | Spoke participants self-report the ECHO network's focus area is relevant to their personal/professional interest at the point of initial registration to join the network. | Redundant. Agreement that this content is adequately covered in revised Item 19. |
| 21 | Spoke participants confirm that ECHO Network topics respond to demonstrated interests that spoke participants identified during learning needs assessments, leading to consistent participant recruitment, and high engagement/attendance in sessions. | Redundant. Agreement that this content is adequately covered in revised Item 19. |
| 23 | Relevance of the case presentation and recommendations content to the wider spoke participants' local practice/role/location is clear. | Redundant. Agreement that this content is adequately covered in revised Item 19. |
| 39 | Interactivity helps to build community of practice that allows for vulnerability and learning. | Redundant. Agreement that this content is adequately covered in revised Item 42. |
| 40 | Spoke participant attendance is regular to build rapport with other participants, such that a community of practice forms/grows. | Redundant. Agreement that this content is adequately covered in revised Item 1. |
| 42 | Optimal size of spoke participant numbers in teleECHO sessions may vary but such that interactivity is not impaired. | Redundant. Agreement that this content is adequately covered in revised Item 37. |
| 83 | Measurement of how well the proposed implementation addresses the identified problem(s), takes advantage of the opportunities identified by using the ECHO model, how it addresses the learning and cultural objectives of the spoke participants and principles of adult learning during the learning needs assessment(s)/planning phase. | Redundant. Agreement that this content is adequately covered in Items 17 and 19. |
| 90 | ECHO Hub teams indicate satisfaction that the ECHO model is valuable in a significantly different way to alternatives. | Redundant. Agreement that this content is adequately covered in revised Item 46. |
| 96 | Spoke participant identifying a number of actual reported changes in their practice or thinking as a result in participation in ECHO sessions. | Redundant. Agreement that this content is adequately covered in revised Item 32. |
| 99 | Measurable change in systems integration attributable to teleECHO Network(s). | Redundant. Difficult to attribute to teleECHO Networks exclusively. |
| 108 | ECHO becomes normalised within the service delivery context of any sector in which it is used. | Redundant. Difficult to attribute to teleECHO Networks exclusively. |

***Appendix 8:***

*Retained Indicators (N=8)*

| # | Indicator of Success |
| --- | --- |
| 12 | Number of spoke participants who present cases for discussion. |
| 27 | Measurable increase in spoke participant's confidence to manage cases locally. |
| 28 | Measurable increase in spoke participant's competence to manage cases locally. |
| 29 | Measurable increase in spoke participant's knowledge/skills to manage cases locally. |
| 30 | Measurable increase in spoke participant's capacity to manage cases locally. |
| 33 | Measurable increase in spoke participant self-efficacy. |
| 34 | Measurable reduction in spoke participant's sense of professional isolation. |
| 35 | Measurable increase in spoke participant's joy of work. |
